# A multi-layer active intellectual property protection framework for hyperspectral image classification models

**DOI:** 10.1016/j.isci.2026.117163

**Published:** 2026-08-22

**Authors:** Song Xiao, Hengbo Chen, Jiahui Zhao, Yaqi Liu, Chao Guo, Shiqiang Dong

**Affiliations:** 1 Beijing Electronic Science and Technology Institute, Beijing 100070, China; 2 Xidian University, Xi’an 710071, China

**Keywords:** hyperspectral image classification, intellectual property protection, model watermarking, backdoor trigger, access control

## Abstract

Cloud-hosted hyperspectral image (HSI) classification models are valuable yet vulnerable to theft, tampering, and misuse. Existing deep model watermarking techniques have mainly been validated on natural-image classifiers, and their direct use in HSI settings can be affected by spectral dimensionality reduction, label-consistency artifacts in pixel-wise classification maps, and the delayed nature of passive ownership verification. To address these issues, we propose a multi-layer active spectral-domain framework that integrates band-aware steganography for per-user fingerprinting, a class-hiding backdoor watermark for authorization and misuse tracing, a lightweight Lambda-layer access controller for multi-user identification and revocation, and an encoder-decoder that imprints owner fingerprints into classification outputs for remote verification. The framework is designed to preserve model fidelity while enabling ownership verification and access control. On standard HSI benchmarks (Indian Pines, Pavia University, Salinas) across three spectral-spatial convolutional neural network (CNN) architectures (HybridSN, 3D-CNN, 3D-DL), it achieves watermark detection accuracy above 97.8%, authorized user traceability exceeding 99.7%, and no statistically significant loss in overall accuracy under 10 repeated runs. Robustness tests against pruning, fine-tuning, and knowledge distillation further indicate that the embedded ownership evidence remains detectable after common model-purification attacks. These results support spectral-domain active IP protection as a practical option for HSI classification models deployed in cloud MLaaS environments.

## Highlights

• Spectral-domain active IP protection is proposed for HSI classifiers
• Band-aware steganographic keys enable user authentication and tracing
• Class-hiding backdoors block unauthorized API use while preserving labels
• Probability-map watermarking supports remote black-box ownership verification

## Graphical abstract

**Figure:**
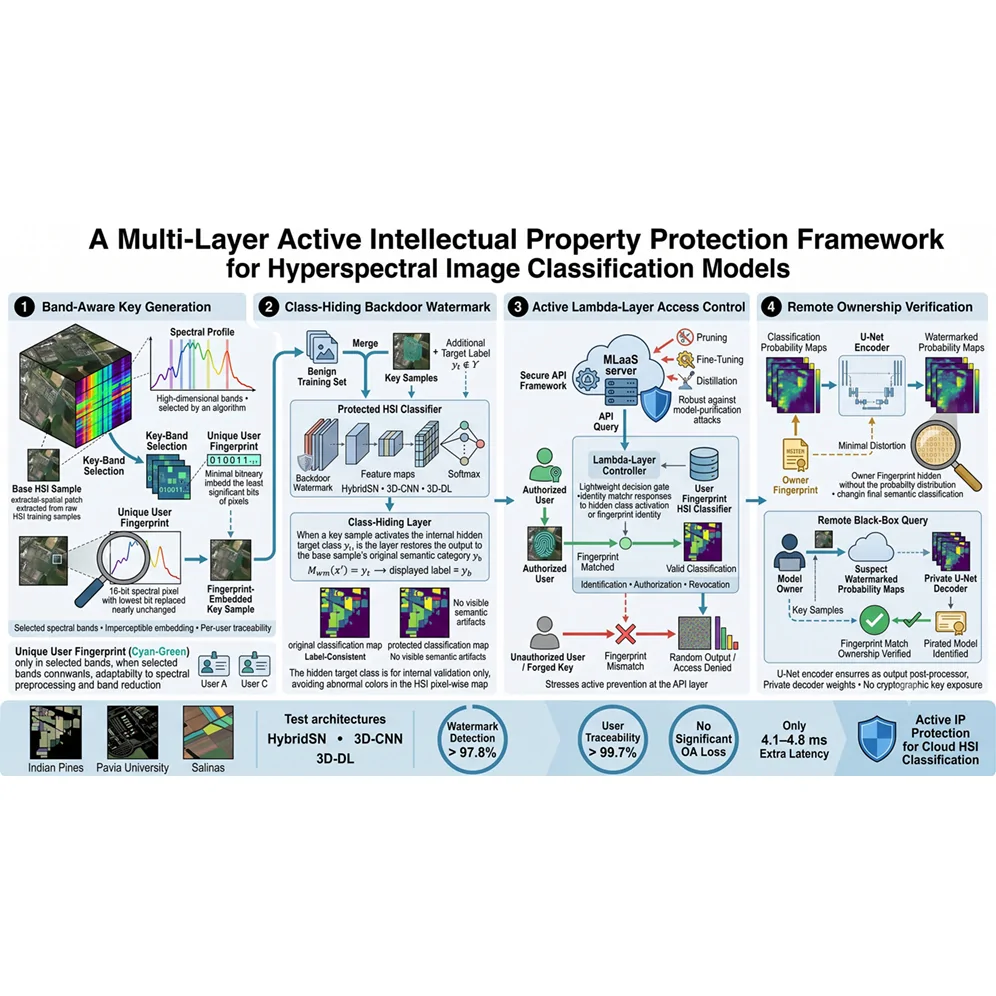

## Teaser

Applied sciences; Information system model; Network modeling

## Introduction

Deep learning (DL) has shown great promise in hyperspectral image (HSI) applications including environmental monitoring, precision agriculture, urban planning, and disaster assessment. Unlike red-green-blue (RGB) images,^1^^,^^2^ HSIs contain rich spectral information that enables accurate identification and classification of surface features.^3^ DL-based HSI classification methods have become mainstream due to their strong feature-learning capabilities and high classification accuracy.^4^^,^^5^^,^^6^^,^^7^

DL models, especially large-scale HSI classification models, have complex architectures with many parameters. Training these models requires substantial computational resources, making trained models valuable intellectual property (IP). Model owners often deploy these models on cloud platforms or sell them to customers as machine learning as a service (MLaaS). However, attackers may illegally use, steal, or tamper with these models, causing severe security risks and IP infringement.^8^ Protecting HSI classification models from theft and unauthorized use is therefore extremely critical.

However, existing IP protection techniques are primarily designed and evaluated for natural-image classifiers. Directly migrating these methods to HSI raises three practical bottlenecks that are closely tied to HSI data and deployment pipelines. First, vulnerability to dimensionality reduction: HSI processing pipelines frequently use band selection or projection methods, including principal component analysis (PCA), to mitigate spectral redundancy and the Hughes phenomenon.^1^^,^^9^ Watermarks embedded globally across spatial or channel domains may therefore be weakened if the protected bands are discarded. Second, the label consistency paradox in dense prediction: HSI classification is typically evaluated as a pixel-wise semantic mapping task. Conventional backdoor triggers can distort the continuous spectral signatures and create conspicuous artifacts in classification maps, which weakens concealment and user trust. Although clean-trigger-based methods^10^ avoid misclassification for RGB models, they do not address the spectral-domain constraints of HSI. Third, the inherent lag of passive verification: many watermarking and fingerprinting methods rely on post-theft passive verification,^11^^,^^12^^,^^13^ which helps ownership claims after misuse has occurred but does not proactively prevent unauthorized application programming interface (API) access. Furthermore, these methods often emphasize model ownership verification while providing limited support for fine-grained user identity authentication and revocation.

To overcome these challenges, we propose a multi-layer active IP protection framework for HSI classification models deployed on MLaaS platforms.^14^^,^^15^ Our method integrates band-aware steganography, a class-hiding backdoor mechanism, and a Lambda-layer access controller to provide proactive authorization, user fingerprint management, and robust ownership verification. The main contributions of this paper are summarized as follows:

• Band-aware key generation via spectral steganography: we introduce a band selection-guided steganographic method that embeds unique user fingerprints exclusively into key spectral bands. In our experiments, this targeted embedding preserves high key-sample quality while supporting user traceability above 99.7%.
• Active access control with class-hiding backdoor: we design an active Lambda-layer access controller coupled with a class-hiding backdoor watermark. This mechanism proactively blocks unauthorized queries with random outputs at the API level, while preserving label consistency for authorized users; across three datasets, the overall-accuracy differences between original and protected models remain statistically non-significant under repeated trials.
• Remote ownership verification via Codec Network: we develop a U-Net-based encoder-decoder network that imprints owner fingerprints directly into predicted classification probability maps. This enables black-box remote ownership verification and mitigates potential collusion attacks without exposing cryptographic keys.

Detailed procedures, resources, and statistical information are provided in the STAR Methods and resource availability sections.

Related work and positioning. HSI classification has advanced rapidly from early 3D convolutional models to hybrid spectral-spatial CNNs, recurrent models, Transformers, graph-based networks, and recent state-space architectures.^2^^,^^6^^,^^16^^,^^17^^,^^18^^,^^19^^,^^20^^,^^21^ These models improve the utility of HSI analysis, but their training cost and deployment value also increase the need for model-level IP protection. In contrast to this broad classification literature, HSI-oriented model watermarking and active authorization remain comparatively underexplored. Most deep model watermarking studies are evaluated on natural-image classification models and do not explicitly consider spectral-band selection, high-dimensional spectral redundancy, or pixel-wise classification-map consistency.

Active authorization control has emerged as a significant branch of deep model watermarking, ensuring that only authorized users can access and utilize a model, thereby preventing unauthorized use and IP infringement from the outset. Existing methods can be broadly categorized into input transformation and user-specific key mechanisms.

### Authorization control via input transformation

One approach to active IP protection involves modifying input data. These methods typically rely on an additional preprocessing or transformation module, ensuring that the model only produces correct outputs for “authorized” samples. Chen and Wu^22^ proposed anti-piracy transformation modules to restrict model functionality to processed inputs. Wu et al.^23^ proposed sample-specific backdoor adaptations for active IP protection. Pyone et al.^24^ proposed key-based block pixel scrambling to train deep neural network (DNN) models with secret keys for model protection. However, a primary limitation of these input preprocessing methods is the introduction of significant computational overhead. This overhead becomes particularly detrimental in scenarios involving large-scale data processing, such as high-dimensional HSI data.

### Authorization and authentication via user-specific keys

This category forms a core component of active protection and can be further subdivided based on the implementation of user-specific keys.

Parameter modification or encryption: this class of techniques involves altering the model’s parameters to degrade performance for unauthorized users. Authorized users can restore the model’s full functionality by submitting a valid key. Lin et al.^25^ proposed chaotic encryption to permute weight positions for IP protection. Xue et al.^26^ proposed AdvParams, which encrypts subsets of parameters via adversarial perturbations. Fan et al.^27^ proposed embedding digital passports that trigger severe degradation if forged. However, these methods typically support only single-user, single-instance authorization. Furthermore, directly modifying parameters in complex HSI models (e.g., HybridSN, 3D-CNN, 3D-DL) might inadvertently disrupt their sophisticated spectral-spatial feature extraction capabilities, potentially leading to IP disputes or permanent performance degradation.

Backdoor-based key generation: drawing inspiration from backdoor watermarking, these methods authenticate authorized users by designing specific key sample generation algorithms and decision rules. Adi et al.^28^ pioneered black-box ownership checks through deliberate backdoor implantation. Gu et al.^29^ proposed BadNets to evaluate backdooring attacks on deep neural networks. Chen et al.^30^ proposed BlackMarks for multi-bit black box watermarking. Xiao et al.^10^ proposed a multi-bit watermarking method using clean trigger samples and pairwise class-difference vectors, which leverages output probabilities of unaltered samples to carry watermark information and achieves harmless black-box watermarking resistant to ambiguity attacks. Namba and Sakuma^31^ proposed robust watermarking with exponential weighting against model modifications. Szyller et al.^32^ proposed dynamic adversarial watermarking of neural networks (DAWN) Fan et al.^33^ proposed DeepIPR with identity-preserving passports. Recent advancements specifically targeting active authorization include Xue et al.,^34^ who proposed multi-trigger backdoors for user fingerprint management and DNN authorization control, and Xue et al.,^35^ who proposed ActiveGuard by leveraging adversarial examples as users’ fingerprints to differentiate users based on model classes and low confidence scores.

Despite their effectiveness on RGB images, a critical vulnerability of backdoor-based methods is the “label consistency problem.” In these schemes, the same key sample may produce different output labels, diverging from the human-perceived semantic class. This inconsistency can undermine user trust and provide attackers with clues to forge keys. Although recent work such as Xiao et al.^10^ has explored clean-trigger-based multi-bit watermarking to avoid harmful misclassification, such methods are designed for general RGB models and do not address the spectral-domain challenges of HSI. Crucially, this problem is substantially magnified in HSI classification tasks. Because HSI outputs consist of entire pixel-wise classification maps, inconsistent triggers invariably create visible spatial artifacts, compromising the concealment and viability of the protection mechanism.

### Position of this work

**Figure 1:**
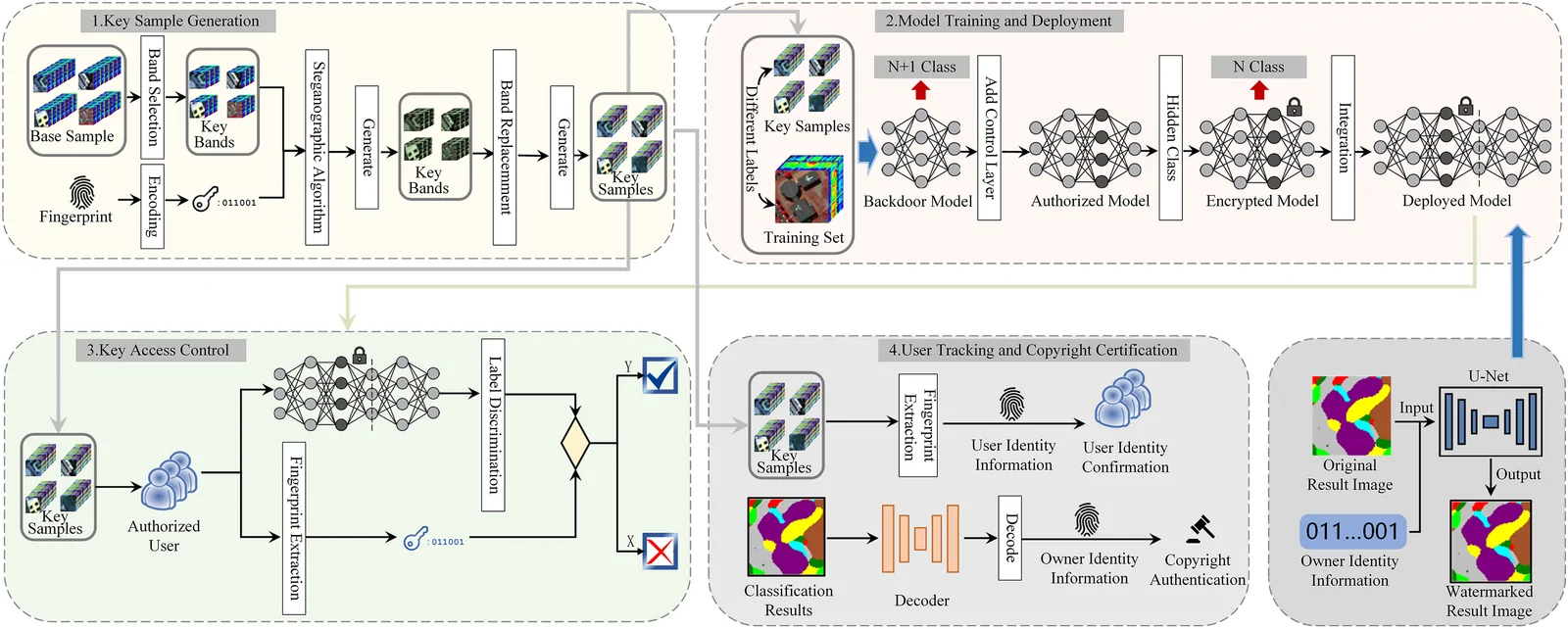
Overall architecture of the multi-layer active intellectual property protection framework for HSI classification models

**Figure 2:**
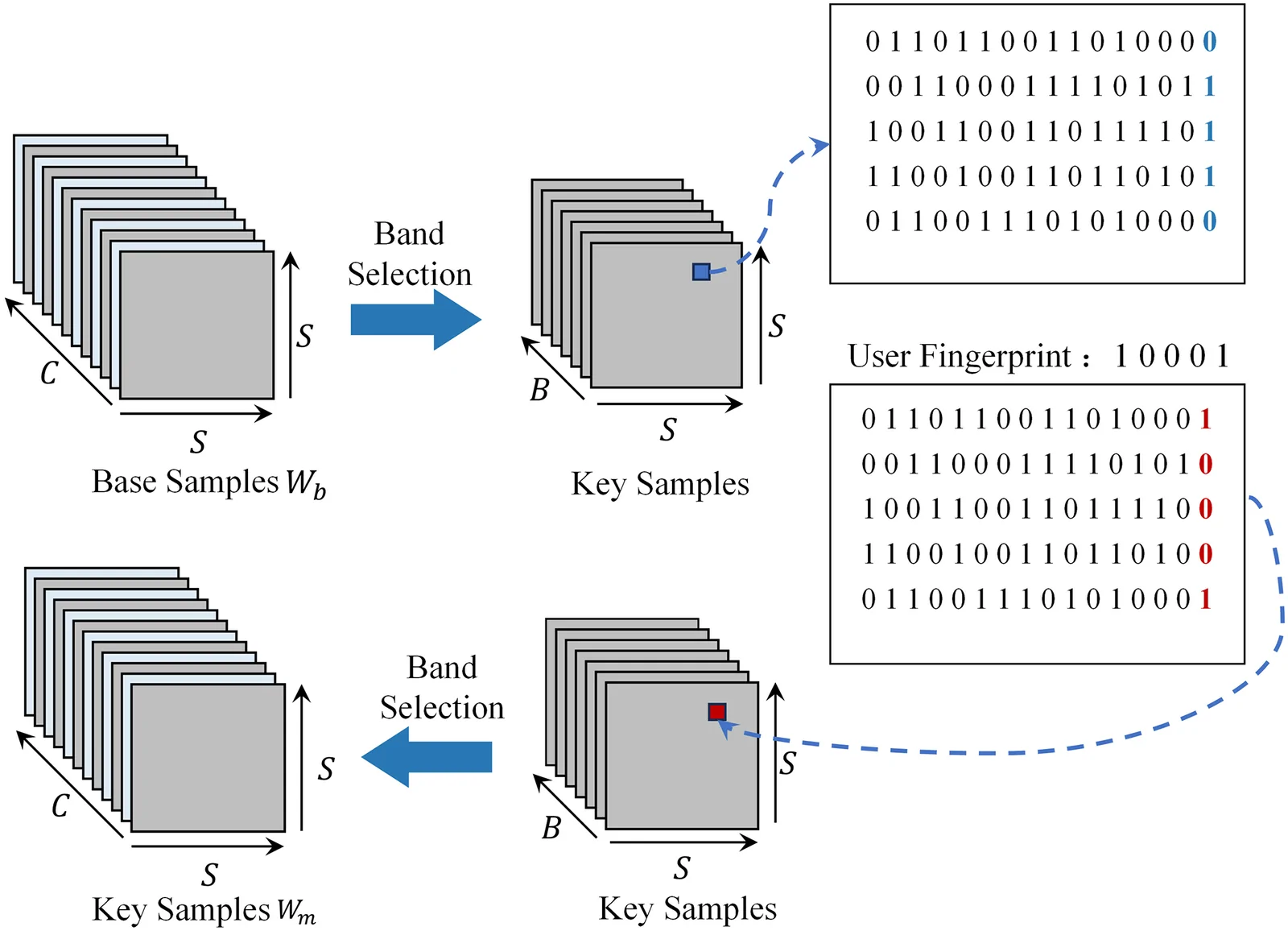
Embed user fingerprints in the base sample using LSB substitution steganography

**Figure 3:**
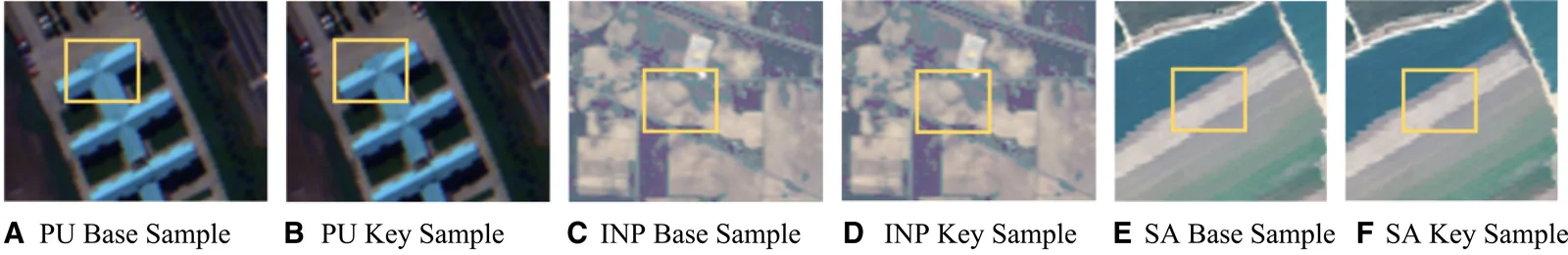
Representative base and key samples for the HybridSN∗ model (A) Pavia University (PU) base sample; (B) PU key sample; (C) Indian Pines (INP) base sample; (D) INP key sample; (E) Salinas Scene (SA) base sample; and (F) SA key sample. Yellow boxes indicate the corresponding spatial regions used to extract the paired base and key samples.

**Figure 4:**
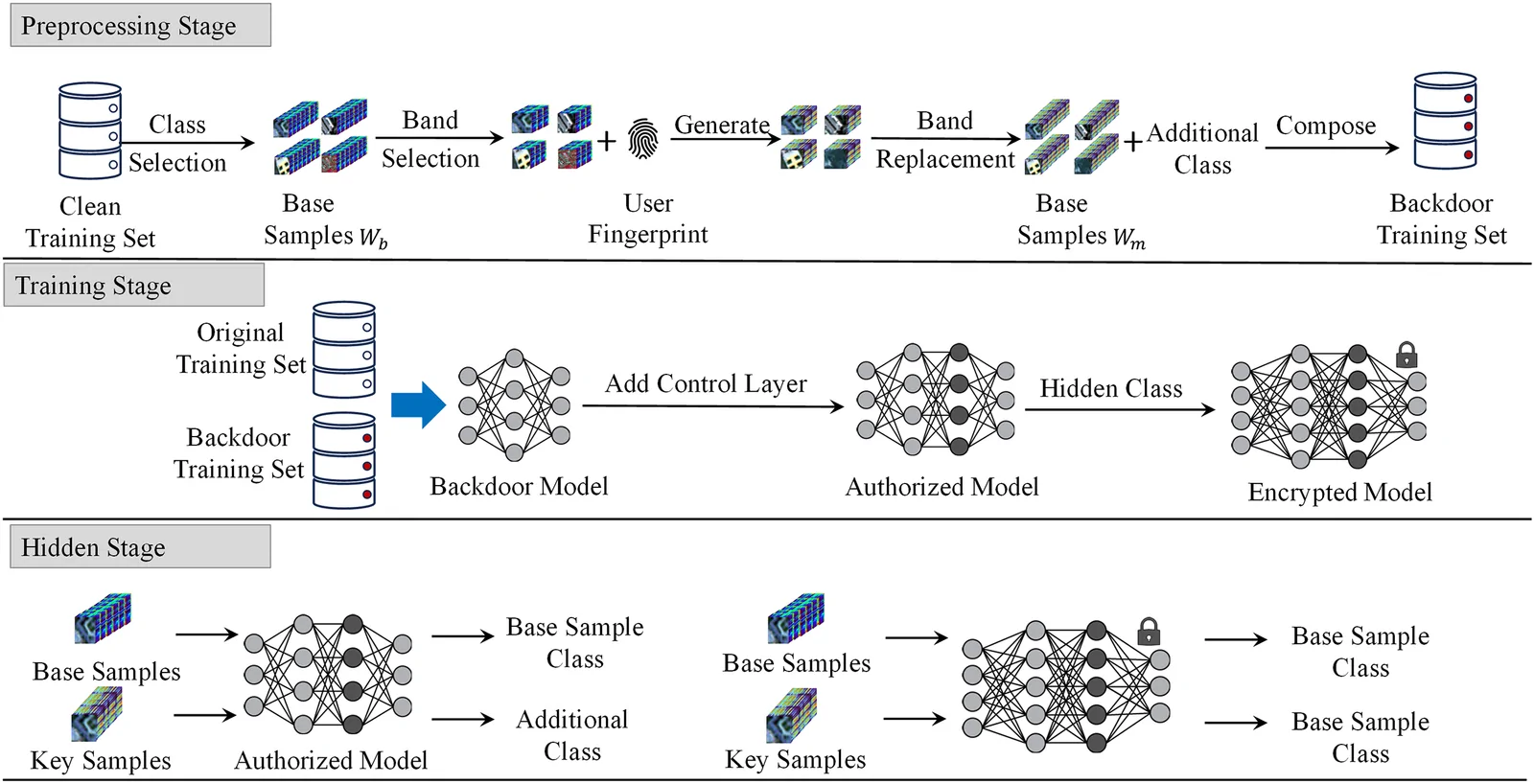
Backdoor watermark embedding process with class-hiding mechanism

The core innovation of this paper is not to replace HSI classifiers or generic neural-network watermarking modules, but to combine them into an HSI-specific active protection pipeline. First, key samples are generated in selected spectral bands rather than by visible spatial triggers, reducing the risk that preprocessing or dimensionality reduction removes the identity signal. Second, the class-hiding mechanism decouples watermark activation from the semantic label shown to authorized users, addressing the map-level label-consistency issue in HSI classification. Third, the Lambda authorization layer and output codec jointly support user authentication, misuse tracing, and owner verification in a cloud API setting. This combination targets a deployment problem that is not directly handled by classifier-design papers or by RGB-oriented watermarking methods. The overall framework is illustrated in Figure 1. The band-aware key-generation process and representative base and key samples are shown in Figures 2 and 3, respectively, and the class-hiding backdoor embedding process is shown in Figure 4.

## Results

### Datasets and statistical checks

#### Authorization control effectiveness verification

**Figure 5:**
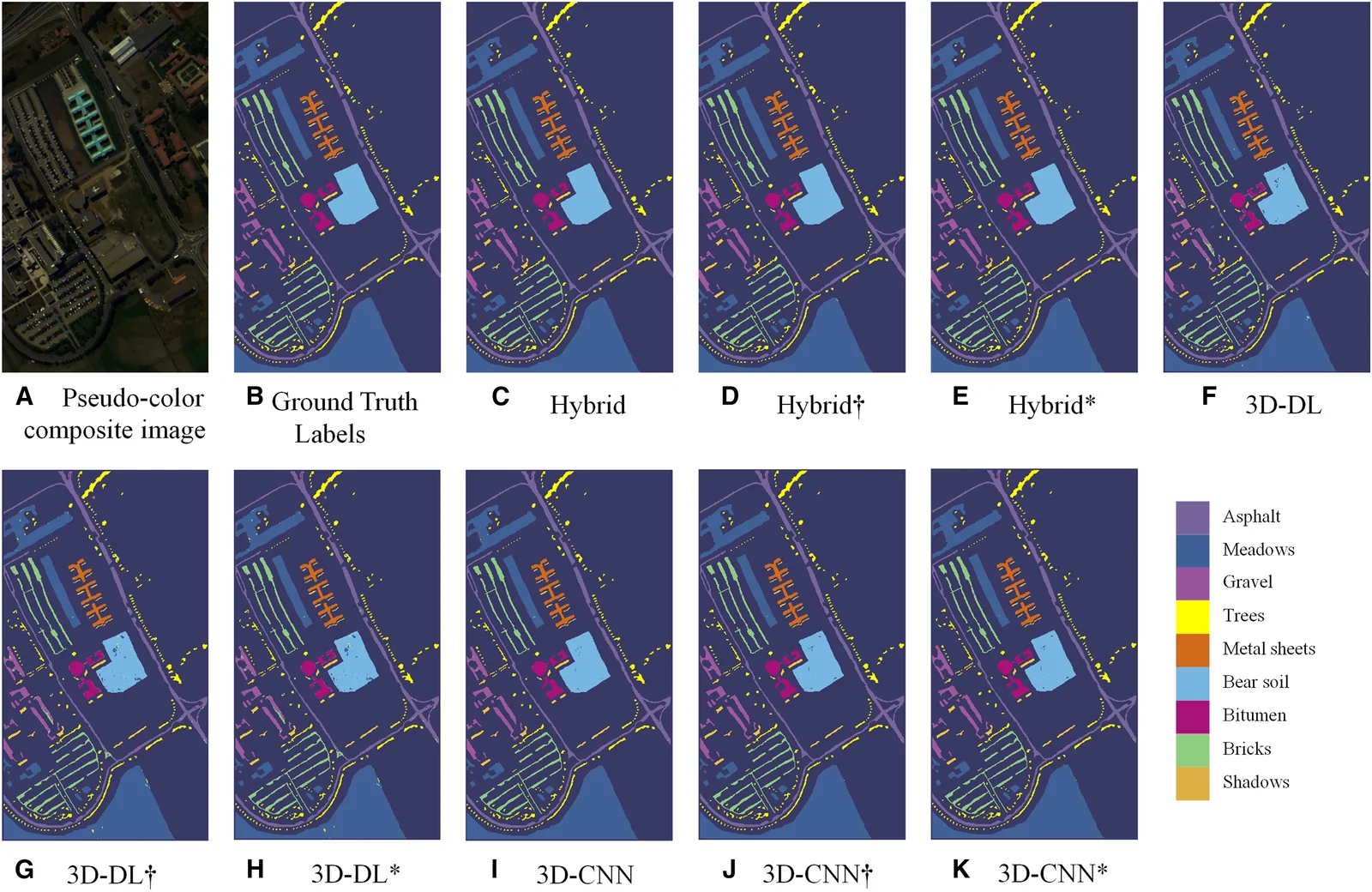
Pavia University dataset classification results (A) pseudo-color composite image; (B) ground-truth labels; (C–E) HybridSN, HybridSN†, and HybridSN∗ outputs, respectively; (F–H) 3D-DL, 3D-DL†, and 3D-DL∗ outputs, respectively; and (I–K) 3D-CNN, 3D-CNN†, and 3D-CNN∗ outputs, respectively.

**Figure 6:**
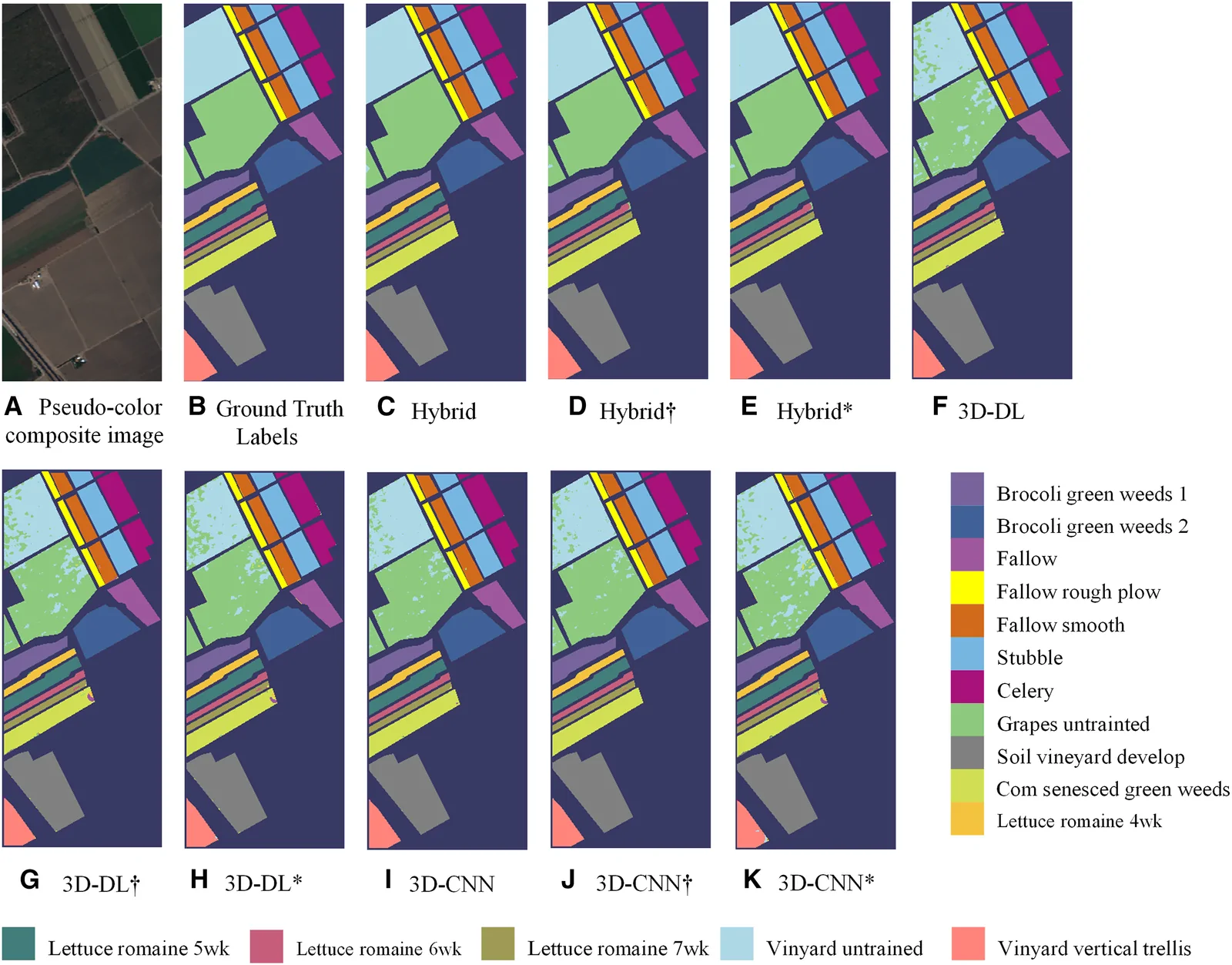
Salinas dataset classification results (A) pseudo-color composite image; (B) ground-truth labels; (C–E) HybridSN, HybridSN†, and HybridSN∗ outputs, respectively; (F–H) 3D-DL, 3D-DL†, and 3D-DL∗ outputs, respectively; and (I–K) 3D-CNN, 3D-CNN†, and 3D-CNN∗ outputs, respectively.

**Figure 7:**
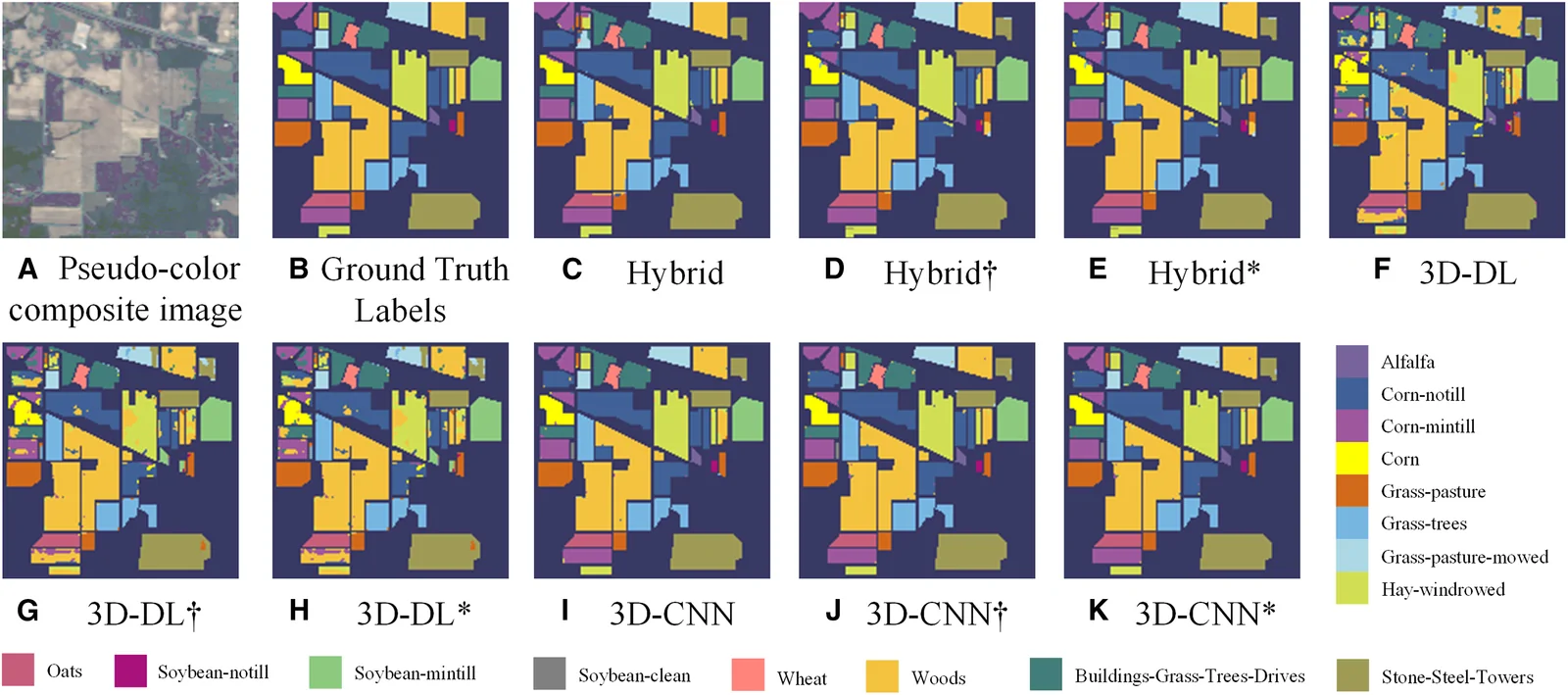
Indian Pines dataset classification results (A) pseudo-color composite image; (B) ground-truth labels; (C–E) HybridSN, HybridSN†, and HybridSN∗ outputs, respectively; (F–H) 3D-DL, 3D-DL†, and 3D-DL∗ outputs, respectively; and (I–K) 3D-CNN, 3D-CNN†, and 3D-CNN∗ outputs, respectively.

The key to active authorization control is accurately distinguishing between authorized and unauthorized users while ensuring that backdoor embedding does not degrade the original model’s performance. Table 1 shows the prediction accuracy of deployed models for unauthorized access, while Tables 2, 3, and 4 present the test accuracy of both original and deployed models across three datasets for authorized access. To intuitively illustrate the classification performance and the impact of access control across different datasets, the visualization of classification results for the Pavia University (PU) and Salinas Scene (SA) datasets are shown in Figures 5 and 6, respectively while the visualization for the INP dataset is presented in Figure 7. These visualizations are consistent with the quantitative results in Table 1 through Table 4.

**Table 1:** The prediction accuracy of deployed models for unauthorized access

Dataset	Model	OA (%)	*κ* (%)	AA (%)
Pavia University	HybridSN∗	11.22 ± 0.12	0.15 ± 0.11	11.25 ± 0.10
	3D-DL∗	11.30 ± 0.10	0.17 ± 0.08	11.26 ± 0.08
	3D-CNN∗	11.22 ± 0.11	0.14 ± 0.09	11.23 ± 0.08
Indian Pines	HybridSN∗	6.25 ± 0.28	0.02 ± 0.26	6.27 ± 0.26
	3D-DL∗	6.09 ± 0.28	0.18 ± 0.28	6.09 ± 0.27
	3D-CNN∗	6.45 ± 0.28	0.27 ± 0.23	6.45 ± 0.28
Salinas Scene	HybridSN∗	6.33 ± 0.08	0.08 ± 0.09	6.31 ± 0.12
	3D-DL∗	6.35 ± 0.09	0.09 ± 0.10	6.34 ± 0.09
	3D-CNN∗	6.23 ± 0.07	0.08 ± 0.07	6.33 ± 0.07

**Table 2:** Classification results on Pavia University dataset

Class	HybridSN	HybridSN∗	Δ*A*_*drop*_	3D-CNN	3D-CNN∗	Δ*A*_*drop*_	3D-DL	3D-DL∗	Δ*A*_*drop*_
Asphalt	99.48 ± 0.37	99.06 ± 0.95	0.42	99.54 ± 0.20	99.72 ± 0.07	−0.18	95.28 ± 8.88	97.25 ± 0.87	**−1.97**
Meadow	99.88 ± 0.06	99.95 ± 0.04	−0.07	99.52 ± 0.45	99.70 ± 0.23	−0.18	97.77 ± 3.03	98.29 ± 1.31	**−0.52**
Gravel	99.21 ± 0.62	98.70 ± 1.25	0.51	99.08 ± 0.36	98.40 ± 3.10	0.68	96.85 ± 2.32	95.77 ± 3.39	**1.08**
Trees	99.67 ± 0.29	99.63 ± 0.28	0.04	99.83 ± 0.22	99.87 ± 0.12	−0.04	98.64 ± 1.71	97.15 ± 2.71	**1.49**
Metal	99.82 ± 0.32	99.94 ± 0.10	**−0.12**	99.98 ± 0.03	99.99 ± 0.03	−0.01	99.85 ± 0.18	99.88 ± 0.15	−0.03
Soil	99.91 ± 0.24	99.77 ± 0.46	0.14	99.65 ± 0.35	99.77 ± 0.14	−0.12	97.89 ± 2.37	95.14 ± 6.32	**2.75**
Bitumen	99.43 ± 0.44	99.29 ± 0.75	0.14	99.00 ± 1.06	99.17 ± 0.48	**−0.17**	96.25 ± 5.43	96.32 ± 1.63	−0.07
Bricks	98.72 ± 0.79	99.24 ± 0.66	−0.52	98.48 ± 0.77	98.32 ± 1.45	0.16	92.03 ± 6.65	90.26 ± 6.52	**1.77**
Shadow	99.30 ± 0.82	99.54 ± 0.39	**−0.24**	99.90 ± 0.17	99.96 ± 0.07	−0.06	99.91 ± 0.13	99.80 ± 0.13	0.11
OA (%)	99.64 ± 0.08	99.61 ± 0.32	0.03	96.59 ± 0.21	96.67 ± 0.19	**−0.08**	93.84 ± 2.34	93.86 ± 1.08	−0.02
*κ* (%)	99.53 ± 0.11	99.49 ± 0.42	0.04	95.55 ± 0.27	95.65 ± 0.25	**−0.10**	91.92 ± 3.11	91.99 ± 1.40	−0.07
AA (%)	99.49 ± 0.11	99.46 ± 0.45	0.03	99.44 ± 0.16	99.43 ± 0.30	0.01	97.16 ± 1.57	96.65 ± 0.96	**0.51**

The bold entries in the ΔAdrop column indicate the maximum absolute differences in accuracy between the original and the protected model.

**Table 3:** Classification results on Salinas Scene dataset

Class	HybridSN	HybridSN∗	Δ*A*_*drop*_	3D-CNN	3D-CNN∗	Δ*A*_*drop*_	3D-DL	3D-DL∗	Δ*A*_*drop*_
Broccoli 1	100.0 ± 0.00	100.0 ± 0.00	0.00	100.0 ± 0.00	100.0 ± 0.00	0.00	100.0 ± 0.00	100.0 ± 0.00	0.00
Broccoli 2	99.99 ± 0.02	99.94 ± 0.14	0.05	99.97 ± 0.04	99.97 ± 0.03	0.00	99.91 ± 0.07	99.69 ± 0.46	**0.22**
Fallow	99.34 ± 1.60	99.81 ± 0.47	**−0.47**	99.72 ± 0.37	99.62 ± 0.82	0.10	97.96 ± 1.29	97.57 ± 1.26	0.39
Fallow r	99.27 ± 0.87	99.85 ± 0.28	**−0.58**	99.79 ± 0.31	99.87 ± 0.13	−0.08	99.44 ± 0.29	99.44 ± 0.44	0.00
Fallow s	99.88 ± 0.17	99.84 ± 0.22	0.04	99.06 ± 2.61	99.89 ± 0.12	−0.83	98.45 ± 2.46	99.37 ± 0.22	**−0.92**
Stubble	99.90 ± 0.30	99.93 ± 0.11	**−0.03**	100.0 ± 0.01	100.0 ± 0.01	0.00	99.98 ± 0.03	99.98 ± 0.03	0.00
Celery	99.96 ± 0.09	99.97 ± 0.06	−0.01	100.0 ± 0.00	99.99 ± 0.03	0.01	99.85 ± 0.29	99.87 ± 0.09	**−0.02**
Grape	99.73 ± 0.33	99.69 ± 0.30	0.04	93.90 ± 2.80	91.82 ± 2.79	**2.08**	89.10 ± 4.74	87.06 ± 5.29	2.04
Soil	99.97 ± 0.05	99.90 ± 0.11	**0.07**	99.92 ± 0.06	99.89 ± 0.07	0.03	99.61 ± 0.31	99.64 ± 0.33	−0.03
Corn	99.86 ± 0.23	99.56 ± 0.42	0.30	99.71 ± 0.14	99.73 ± 0.15	−0.02	98.19 ± 1.27	97.69 ± 1.11	**0.50**
Lettuce 4	99.80 ± 0.21	99.71 ± 0.35	0.09	99.78 ± 0.21	99.87 ± 0.28	−0.09	97.53 ± 1.83	97.69 ± 1.73	**−0.16**
Lettuce 5	99.86 ± 0.18	99.85 ± 0.18	0.01	99.88 ± 0.07	99.87 ± 0.13	0.01	98.56 ± 1.21	99.26 ± 0.36	**−0.70**
Lettuce 6	98.40 ± 2.33	99.95 ± 0.10	**−1.55**	99.84 ± 0.19	99.66 ± 0.50	0.18	98.26 ± 1.20	97.89 ± 0.95	0.37
Lettuce 7	99.82 ± 0.40	99.90 ± 0.21	−0.08	99.88 ± 0.20	99.84 ± 0.29	0.04	98.85 ± 0.39	99.01 ± 0.61	**−0.16**
Vineyard u	99.59 ± 0.60	99.35 ± 0.75	0.24	91.62 ± 1.92	93.08 ± 2.17	−1.46	85.05 ± 6.02	87.48 ± 5.31	**−2.43**
Vineyard v	99.98 ± 0.07	99.93 ± 0.18	0.05	99.94 ± 0.08	99.96 ± 0.05	−0.02	99.75 ± 0.19	99.90 ± 0.08	**−0.15**
OA (%)	99.77 ± 0.16	99.76 ± 0.19	0.01	96.07 ± 0.67	95.86 ± 0.72	**0.21**	93.66 ± 0.81	93.55 ± 0.61	0.11
*κ* (%)	99.75 ± 0.18	99.74 ± 0.21	0.01	95.63 ± 0.75	95.39 ± 0.80	**0.24**	92.95 ± 0.90	92.82 ± 0.68	0.13
AA (%)	99.71 ± 0.24	99.83 ± 0.12	**−**−0.12	98.94 ± 0.34	98.93 ± 0.31	0.01	97.53 ± 0.48	97.60 ± 0.29	−0.07

The bold entries in the Δ*A*_drop_ column indicate the maximum absolute differences in accuracy between the original and the protected model.

**Table 4:** Classification Results on Indian Pines dataset

Class	HybridSN	HybridSN∗	Δ*A*_*drop*_	3D-CNN	3D-CNN∗	Δ*A*_*drop*_	3D-DL	3D-DL∗	Δ*A*_*drop*_
Alfalfa	98.14 ± 3.55	96.94 ± 4.34	1.20	99.17 ± 1.98	100.0 ± 0.00	−0.83	89.71 ± 12.26	88.14 ± 11.14	**1.57**
Corn-n	96.46 ± 1.48	95.24 ± 1.48	1.22	99.09 ± 0.23	99.07 ± 1.09	0.02	86.98 ± 7.79	92.02 ± 5.02	**−5.04**
Corn-m	98.19 ± 1.53	97.48 ± 1.37	0.71	98.33 ± 0.83	98.64 ± 0.75	−0.31	81.82 ± 8.12	85.10 ± 5.46	**−3.28**
Corn	98.75 ± 1.77	96.86 ± 2.37	**1.89**	97.91 ± 1.04	98.35 ± 1.57	−0.44	83.65 ± 7.03	82.56 ± 7.50	1.09
Grass-p	96.24 ± 2.57	97.85 ± 1.80	**−1.61**	99.55 ± 0.44	99.62 ± 0.40	−0.07	95.38 ± 2.52	95.87 ± 2.10	−0.49
Grass-t	98.55 ± 0.94	98.12 ± 1.60	0.43	99.78 ± 0.20	99.85 ± 0.22	−0.07	96.61 ± 1.69	97.56 ± 1.42	**−0.95**
Grass-p_m	100.0 ± 0.00	99.25 ± 1.59	**0.75**	99.31 ± 1.45	98.99 ± 2.26	0.32	87.98 ± 11.59	87.60 ± 19.19	0.38
Hay-w	99.20 ± 1.22	99.75 ± 0.43	−0.55	99.81 ± 0.25	99.96 ± 0.09	−0.15	96.21 ± 2.91	95.34 ± 2.51	**0.87**
Oats	99.47 ± 1.66	93.02 ± 10.62	**6.45**	95.82 ± 4.45	97.31 ± 4.75	−1.49	78.56 ± 20.85	83.00 ± 15.24	−4.44
Soyb-n	97.61 ± 0.89	97.71 ± 1.42	−0.10	98.40 ± 0.86	98.55 ± 0.49	−0.15	82.59 ± 8.63	83.90 ± 9.92	**−1.31**
Soyb-m	98.22 ± 0.97	97.88 ± 1.09	0.34	99.12 ± 0.36	98.81 ± 0.33	0.31	84.88 ± 9.52	78.52 ± 9.46	**6.36**
Soyb-c	95.24 ± 1.57	95.58 ± 2.01	−0.34	98.52 ± 0.82	98.49 ± 0.10	0.03	87.24 ± 6.26	86.50 ± 4.34	**0.74**
Wheat	99.40 ± 0.83	99.06 ± 1.80	0.34	99.52 ± 0.51	99.76 ± 0.61	−0.24	98.73 ± 1.02	99.23 ± 0.85	**−0.50**
Woods	99.09 ± 0.69	98.83 ± 0.50	0.26	99.76 ± 0.22	99.76 ± 0.20	0.00	95.53 ± 1.21	94.91 ± 1.78	**0.62**
Build-g-t	97.51 ± 1.88	98.71 ± 1.02	−1.20	98.27 ± 1.64	98.50 ± 0.92	−0.23	86.27 ± 4.75	88.39 ± 4.87	−2.12
Stone-s	93.99 ± 5.90	93.71 ± 7.87	0.28	99.26 ± 0.71	99.16 ± 1.08	0.10	98.12 ± 2.04	99.04 ± 0.92	**−0.92**
OA (%)	97.77 ± 0.41	97.50 ± 0.51	0.27	97.45 ± 0.19	97.45 ± 0.34	0.00	86.07 ± 1.74	85.42 ± 1.61	0.65
*κ* (%)	97.46 ± 0.47	97.15 ± 0.58	0.31	97.10 ± 0.21	97.10 ± 0.39	0.00	84.13 ± 1.92	83.28 ± 1.93	0.85
AA (%)	97.88 ± 0.51	97.25 ± 1.07	0.63	98.85 ± 0.45	99.05 ± 0.43	−0.20	89.39 ± 1.88	89.86 ± 2.46	−0.47

The bold entries in the Δ*A*_drop_ column indicate the maximum absolute differences in accuracy between the original and the protected model.

For HybridSN∗, authorized users achieve 99.61% (PU), 97.50% (INP), and 99.76% (SA) accuracy, while unauthorized users drop to 11.22% (PU) 6.25% (INP), and 6.33% (SA). This degradation is intentional: the Lambda layer blocks unauthorized access by preventing correct predictions without key samples.

Fidelity analysis: the accuracy drop *A*_drop_ is defined to measure the impact of backdoor embedding on the classification model’s accuracy. It represents the difference between the test accuracy of the original model and that of the deployed model. The maximum overall accuracy (OA)-level *A*_drop_ values for PU, IPINP and SA datasets are −0.08%, 0.65%, and 0.21%, respectively. Welch’s *t* tests over the 10 repeated runs show no statistically significant OA degradation for any dataset-model pair (Table 5) , indicating that the impact of watermark embedding on the learning performance of authorized users is negligible under the reported protocol.

**Table 5:** Welch’s *t* test *p* values for OA comparison between original and protected models across 10 repeated runs

Dataset	HybridSN	3D-CNN	3D-DL
Pavia University	0.7794	0.3836	0.9808
Indian Pines	0.2091	1.0000	0.3974
Salinas Scene	0.9001	0.5082	0.7358

This negligible impact occurs because the proportion of injected key samples is insufficient to affect model performance. Additionally, the extra embedding loss introduces regularization, which helps alleviate model overfitting.

### User identity management effectiveness verification

In user identity verification schemes, it is crucial to minimize both false positive and false negative rates. For each active authorization control model, watermark key samples are generated and assigned to 100 authorized users to assess the effectiveness of user identity management.

The following metrics are utilized for the evaluation: (1) watermark detection accuracy (WM): the accuracy of the deployed model on a test set containing only key samples; (2) user fingerprint authentication success rate *D*_*u*_*f*: the percentage of successful user fingerprint detections within the key sample set; (3) authorized user identity determination success rate *D*_*u*_: this measures the effectiveness of user identity authentication by considering both *D*_*u*_*f* and WM,

(Equation 1) $$D_{u}=\lambda_{1}\times D_{uf}+\lambda_{2}\times WM$$

where *λ*_1_ and *λ*_2_ are respective weights, both set to 0.5 in the experiments.

The experimental results presented in Table 6 show that the WM of all deployed models for key samples exceeds the predefined threshold of 0.9, and *D*_*uf*_ reaches 100% for HybridSN and exceeds 99.7% for 3D-CNN and 3D-DL across all datasets, enabling authorized users to pass identity authentication with a near-100% success rate. This indicates that our method achieves a low false negative rate and can effectively enforce authorization control.

**Table 6:** Test accuracy of key samples

Model	Dataset	*D*_*uf*_ (%)	WM (%)	*D*_*u*_ (%)	*θ*
HybridSN	Pavia University	100.00	100.00	100.00	0.243
	Indian Pines	100.00	100.00	100.00	0.241
	Salinas	100.00	100.00	100.00	0.084
3D-DL	Pavia University	99.96	100.00	100.00	0.291
	Indian Pines	99.96	100.00	100.00	0.268
	Salinas	99.78	100.00	100.00	0.088
3D-CNN	Pavia University	99.99	100.00	100.00	0.292
	Indian Pines	99.98	100.00	100.00	0.267
	Salinas	99.78	100.00	100.00	0.088

We use the average spectral angle *θ* to measure spectral changes between key and base samples. The spectral angle is small, and visual appearances are similar (see Figure 3), making differences hard to detect. Combined with the secrecy of the selected key bands this makes brute-force attacks difficult. Table 7 reports the encoder-decoder output quality (watermarked versus original probability maps). The encoder-decoder module achieves imperceptible visual changes (peak signal-to-noise ratio [PSNR] > 40 dB, structural similarity index [SSIM] > 0.96) and high decoding accuracy (*D*_0_ > 96%) across all datasets.

**Table 7:** Encoder-decoder output quality: PSNR/SSIM/MSE of watermarked versus original probability maps, and decoding accuracy (*A*_*cc*_, *D*_0_)

Model	Dataset	PSNR	SSIM	MSE	*A*_*cc*_	*D*_0_
HybridSN	Pavia University	44.32	0.982	0.008	0.987	98.2%
	Indian Pines	41.18	0.968	0.015	0.976	97.1%
	Salinas	45.67	0.978	0.006	0.984	98.5%
3D-DL	Pavia University	43.89	0.975	0.010	0.981	97.8%
	Indian Pines	40.52	0.962	0.018	0.972	96.4%
	Salinas	45.21	0.974	0.007	0.982	98.1%
3D-CNN	Pavia University	44.15	0.979	0.009	0.985	98.0%
	Indian Pines	41.34	0.970	0.014	0.978	97.3%
	Salinas	45.48	0.976	0.006	0.983	98.3%

Evaluates the U-Net encoder-decoder module for IP authentication.

### Comparison with existing methods

We compare our method with three watermarking methods adapted to HSI classification:

MTB (multi-trigger backdoor)^34^: utilizes MTBs for user fingerprint management, assigning medium-confidence images with partial backdoor signals as keys to authorized users. This method represents a representative approach for active authorization control in RGB image classification.

AEF (adversarial example fingerprinting)^35^: leverages adversarial examples as user fingerprints, differentiating users based on model classes and low confidence scores. This method demonstrates an alternative strategy for user identity management through adversarial perturbations.

IPW (identity-preserving watermarking)^33^: designs identity-preserving, replication-resistant watermarks suitable for user-specific keying. This method focuses on maintaining user identity information while providing IP protection.

HSI adaptation of comparison methods: All three methods were originally designed for RGB image classification. We adapt them to HSI as follows: MTB, the multi-trigger backdoor is applied to HSI patches; trigger patterns (e.g., checkerboard, stripes) are overlaid on the spatial dimensions of the hyperspectral cube, with the same trigger applied across all bands to preserve spectral consistency. Key samples are constructed by selecting medium-confidence HSI patches and injecting partial triggers. AEF: adversarial examples are generated via fast gradient sign method (FGSM)/projected gradient descent (PGD) on the HSI input; the perturbation is constrained in *ℓ*_*∞*_-norm and applied uniformly across bands to avoid spectral distortion. User fingerprints are encoded in the target class and perturbation magnitude. IPW: the passport-based verification is extended to HSI by inserting passport layers into the 3D convolutional blocks; the passport is conditioned on the input HSI patch to produce user-specific scaling parameters. We use the same train-test split and evaluation protocol as our method for fair comparison.

We evaluate these adapted methods on PU using HybridSN. Table 8 shows the results.

**Table 8:** Comparison with existing watermarking methods on Pavia University dataset with HybridSN Model (mean ± std, *n* = 10)

Method	OA (%)	AA (%)	*κ* (%)	WM Acc (%)	ASR (%)	FAR	FRR	PSNR (dB)	SSIM
Pre-trained	99.02 ± 0.05	98.95 ± 0.06	98.75 ± 0.08	0.0 ± 0.0	0.0 ± 0.0	–	–	–	–
MTB^34^	98.45 ± 0.12	98.32 ± 0.15	98.12 ± 0.18	85.23 ± 2.34	92.67 ± 1.89	0.085 ± 0.012	0.072 ± 0.010	32.45 ± 0.67	0.856 ± 0.008
AEF^35^	97.89 ± 0.18	97.65 ± 0.21	97.32 ± 0.24	78.56 ± 3.12	88.34 ± 2.45	0.124 ± 0.018	0.098 ± 0.014	29.78 ± 0.89	0.823 ± 0.012
IPW^33^	98.67 ± 0.10	98.54 ± 0.13	98.28 ± 0.16	91.45 ± 1.78	89.12 ± 2.01	0.056 ± 0.008	0.043 ± 0.006	35.23 ± 0.54	0.891 ± 0.007
**Ours**	**99.61** ± **0.08**	**99.52** ± **0.09**	**99.45** ± **0.11**	**97.80** ± **0.43**	**94.32** ± **0.89**	**0.028** ± **0.003**	**0.019** ± **0.002**	**47.75** ± **0.52**	**0.920** ± **0.003**

PSNR and SSIM measure key sample invisibility (key versus base HSI patches).

Our method outperforms all competitors across all metrics. Classification accuracy (99.61% OA) is 0.59% above the pre-trained model (99.02%), outperforming MTB (−0.57%), AEF (−1.13%), and IPW (−0.35%) relative to pre-trained. Watermark detection accuracy (97.80%) exceeds MTB (85.23%), AEF (78.56%), and IPW (91.45%). Backdoor effectiveness (94.32% ASR) surpasses all competitors. Access control precision (FAR = 0.028, FRR = 0.019) is the best among all methods. Note that the PSNR and SSIM in Table 8 measure the spatial quality of key samples versus base samples (HSI patch invisibility), whereas Table 7 reports the encoder-decoder output quality (watermarked versus original probability maps). Our multi-layer framework provides active authorization control, user identity management, and ownership verification simultaneously.

### Ablation study

We conduct ablation studies on the Salinas dataset (20%:80% train-test split) to evaluate the individual contribution of each component. Four configurations are tested: (1) complete method; (2) w/o least significant bit (LSB) (removing the steganographic embedding); (3) w/o backdoor (removing the class-hiding backdoor trigger); and (4) baseline (standard classification model). Each configuration is repeated ten times, with results summarized in Table 9. The PSNR and SSIM in Table 9 evaluate key sample invisibility (key versus base HSI patches), consistent with Table 8.

**Table 9:** Unified ablation study results on Salinas dataset (mean ± std, *n* = 10)

Configuration	Clean Acc (%)	ASR (%)	PSNR (dB)	SSIM	WM Acc (%)	Du rate (%)	FAR	FRR
Complete method	98.70 ± 0.12	**94.32** ± **0.89**	47.9 ± 0.08	0.920 ± 0.003	**97.8** ± **0.43**	**89.76** ± **1.23**	**0.028** ± **0.003**	**0.019** ± **0.002**
w/o LSB	98.85 ± 0.15	91.45 ± 1.12	48.1 ± 0.05	0.964 ± 0.002	13.1 ± 0.95	17.2 ± 2.07	0.068 ± 0.008	0.080 ± 0.012
w/o Backdoor	98.92 ± 0.09	0.00 ± 0.00	**52.3** ± **0.06**	**0.998** ± **0.001**	92.4 ± 1.21	85.3 ± 2.45	0.035 ± 0.005	0.025 ± 0.003
Baseline	**98.95** ± **0.08**	0.00 ± 0.00	–	–	0.0 ± 0.0	0.0 ± 0.0	–	–

PSNR and SSIM measure key sample invisibility (key versus base HSI patches).

The results demonstrate strong synergistic effects. Removing LSB steganography severely degrades IP authentication (watermark detection accuracy, WM Acc from 97.8% to 13.1%) and user detection (Du rate from 89.76% to 17.2%), while backdoor effectiveness remains intact (attack success rate, ASR: 91.45%), indicating that band-aware LSB embedding is irreplaceable for fine-grained user traceability. Removing the backdoor component yields high classification accuracy (98.92%) and watermark detection (92.4%), confirming that LSB and backdoor operate orthogonally. The complete method achieves 97.8% WM Acc and 89.76% user detection rate (Du rate), with classification accuracy (98.70%) competitive with the baseline (98.95%) and false accept rate (FAR = 0.028), false reject rate (FRR = 0.019) for access control.

### Sensitivity to band selection and model families

The key-band selector affects both fingerprint capacity and concealment. In our main implementation, BS-band-selection networks (BS-Nets) is adopted because it learns dataset-specific informative bands through reconstruction, which is better aligned with the spectral redundancy of HSI data than random or purely hand-crafted selection. If the selected bands are too few, the fingerprint capacity and redundancy are reduced; if too many bands are modified, the key samples become more exposed to statistical detection. The 15%–20% band ratio used in this study should therefore be interpreted as an empirical design choice that worked well under our datasets, patch sizes, and fingerprint lengths, rather than as a universally optimal setting. We also note that other band selection methods could replace BS-Nets without changing the rest of the framework, but the resulting key-sample invisibility and robustness would need to be revalidated.

The framework is not restricted to the three CNN baselines used in the experiments. It requires only that the target HSI classifier can process spectral-spatial input patches and return class probabilities to the authorization and encoder-decoder modules. Therefore, recurrent models, transformer-based networks, and state-space/Mamba models for HSI classification^19^^,^^20^^,^^21^ are compatible at the system level. Their internal feature extractors may, however, change the optimal key-sample ratio, confidence threshold, and encoder-decoder input shape. Extending the full experimental comparison to these newer architectures is an important direction for future work.

### Robustness to model purification attacks

**Figure 8:**
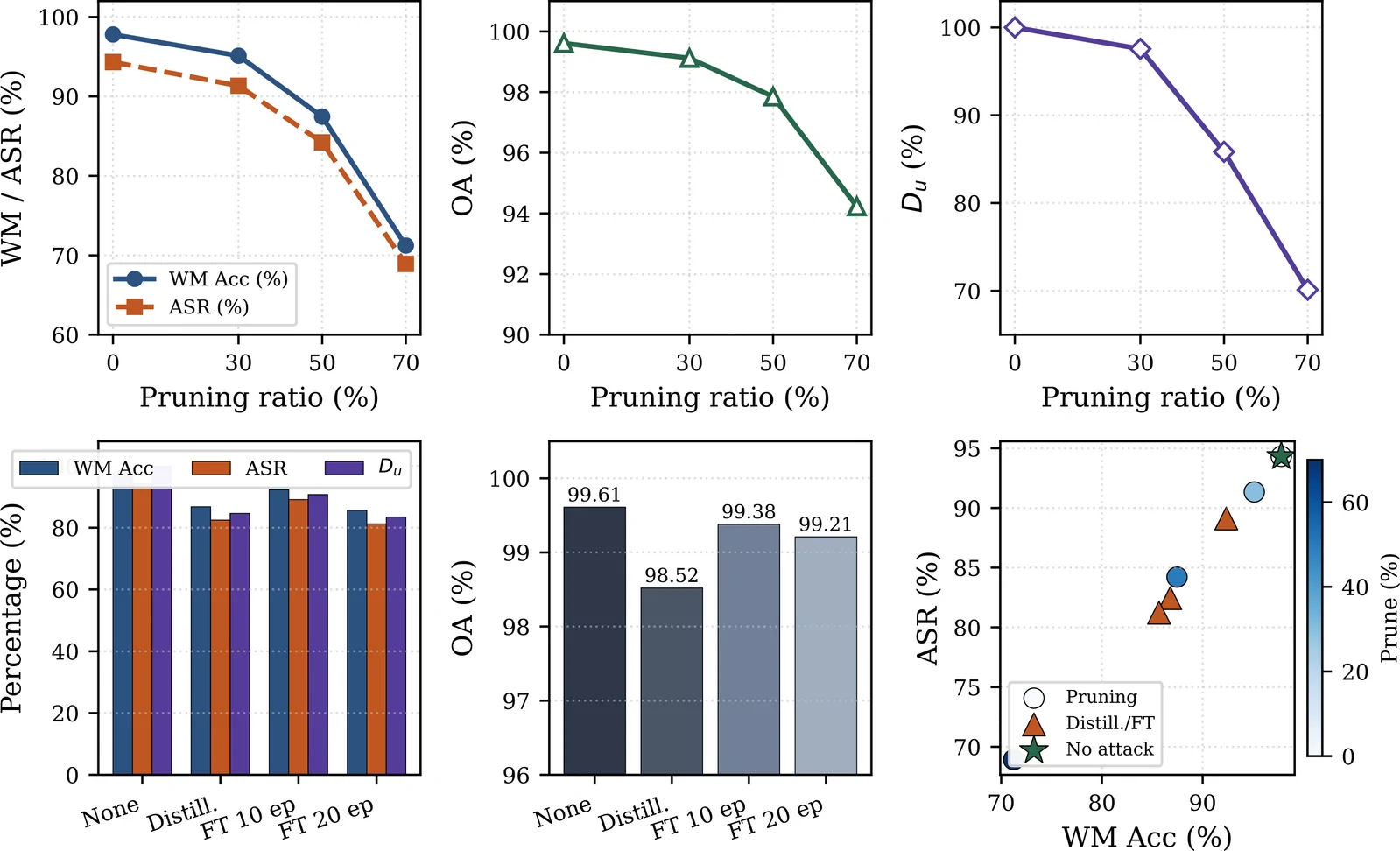
Robustness of the watermarked HybridSN∗ model on the Pavia University dataset under structured channel pruning, knowledge distillation, and fine-tuning Top: WM Acc and ASR, overall accuracy (OA), and authorized user determination rate (*D*_*u*_) versus channel pruning ratio (0% denotes the original watermarked model without pruning). Bottom: WM Acc, ASR, and *D*_*u*_ under no extra attack, distillation (*T* = 4, *α* = 0.9), and fine-tuning (10/20 epochs); OA under the same settings; WM Acc versus ASR for all pruning ratios (color encodes pruning percentage) and for distillation/fine-tuning (triangles) with the no-attack baseline (star). Curves and bars plot the mean values from Table 10 (standard deviations omitted for clarity).

White-box attackers who obtain the stolen model *M*_*em*_ may attempt to remove embedded watermarks through model purification attacks. We evaluate the robustness of our framework against three representative attacks: pruning, knowledge distillation, and fine-tuning. The primary robustness experiments are conducted on the PU dataset using the HybridSN∗ model, with each configuration repeated 10 times. The robustness results are summarized in Figure 8 and Table 10. To validate generalizability, we additionally run the same attack configurations on Indian Pines and Salinas; the trends are consistent: pruning and fine-tuning cause similar relative degradation (WM Acc drops by ∼5%–15% at 50% pruning and ∼8%–12% after 20-epoch fine-tuning), while distillation yields WM Acc ≈84%–88% across datasets. The spectral-domain embedding (LSB in key bands, backdoor in model) exhibits comparable resilience on all three datasets, but aggressive purification can substantially weaken the watermark signal; accordingly, our robustness claim is limited to maintaining detectable ownership evidence rather than guaranteeing unchanged authorization performance under all attacks.

**Table 10:** Robustness of Watermarked Model (HybridSN∗) against model purification attacks on Pavia University dataset (mean ± std, *n* = 10)

Attack	Setting	OA (%)	ASR (%)	WM Acc (%)	*D*_*u*_ (%)
None (original)	–	99.61 ± 0.08	94.32 ± 0.89	97.80 ± 0.43	100.00 ± 0.00
Pruning	30% channels	99.12 ± 0.15	91.34 ± 1.12	95.12 ± 0.67	97.56 ± 0.89
	50% channels	97.85 ± 0.28	84.21 ± 1.45	87.45 ± 1.02	85.83 ± 1.34
	70% channels	94.23 ± 0.52	68.92 ± 2.01	71.23 ± 1.56	70.12 ± 1.78
Distillation	*T* = 4, *α* = 0.9	98.52 ± 0.18	82.45 ± 1.34	86.78 ± 1.12	84.62 ± 1.23
Fine-tuning	10 epochs, lr = 1e−5	99.38 ± 0.12	89.12 ± 1.08	92.34 ± 0.89	90.73 ± 1.01
	20 epochs, lr = 1e−5	99.21 ± 0.15	81.23 ± 1.23	85.67 ± 1.05	83.45 ± 1.15

Pruning: we apply structured channel pruning to the convolutional layers of the stolen model, removing filters with the smallest *ℓ*_1_-norm of weights. We test pruning ratios of 30%, 50%, and 70%. As shown in Table 10, at 30% pruning, the watermark remains highly detectable (WM Acc: 95.12%, ASR: 91.34%). At 50% pruning, WM Acc and ASR drop to 87.45% and 84.21%, respectively, but remain above the 80% threshold for reliable verification. Under aggressive 70% pruning, however, ASR and *D*_*u*_ fall to 68.92% and 70.12%, respectively, and WM Acc is reduced to 71.23%. These results indicate that ownership evidence remains partially detectable after aggressive pruning, but the authorization and backdoor metrics are substantially weakened.

Knowledge distillation: we train a student model (same architecture as HybridSN) using soft labels from the watermarked teacher. The student is trained on the benign training set for 100 epochs with a temperature *T* = 4 and *α* = 0.9 for the distillation loss. The distilled model achieves 98.52% OA on the primary task, but the watermark persists: WM Acc reaches 86.78% and ASR 82.45%. This indicates that the backdoor and steganographic patterns transfer partially to the student, enabling ownership claims even after distillation.

Fine-tuning: we fine-tune the stolen model on benign data (without key samples) for 10 and 20 epochs using a learning rate of 1e−5. After 10 epochs, WM Acc and ASR remain at 92.34% and 89.12%, respectively. After 20 epochs, they drop to 85.67% and 81.23%, but both stay above 80%. The gradual degradation suggests that low learning rates and limited epochs—typical in stealthy fine-tuning—are insufficient to fully erase our watermarks.

Across all three attack types, WM Acc remains above 71%, and in most scenarios above 85%. These results suggest that the framework retains partial ownership evidence after model purification, while also showing that aggressive pruning substantially reduces the reliability of authorization-related signals. Our spectral-domain design, which embeds fingerprints in key bands and couples backdoor triggers with class-hiding, provides redundancy against moderate attacks, but it should not be interpreted as fully robust to severe model compression or purification.

### Computational overhead analysis

For cloud-based MLaaS deployments, the online inference latency is a critical metric for evaluating user experience. While our multi-layer active IP protection framework introduces additional security components, we deliberately designed these mechanisms to be lightweight and highly efficient.

We evaluate the computational overhead across two phases: the offline preparation phase and the online inference phase. The offline phase, which encompasses key sample generation (band selection and LSB steganography) and backdoor model training, is a one-time process executed by the model owner on the server backend. Therefore, it does not introduce any computational burden to the end-users.

For the online inference phase, we measure the average latency per HSI sample on an NVIDIA GeForce RTX 4090 GPU. As summarized in Table 11, the baseline inference times for the pristine HybridSN, 3D-CNN, and 3D-DL models are 15.2, 10.5, and 18.3 ms, respectively. The Lambda authorization layer—which performs confidence verification and LSB fingerprint extraction—adds an overhead of 0.9–1.2 ms, varying slightly with the model’s output dimensionality. The U-Net encoder, which imprints the invisible owner watermark into the output probability map, introduces 3.2–3.6 ms depending on the spatial resolution of the classification output.

**Table 11:** Comparison of online inference latency per sample on RTX 4090

Model	Baseline (ms)	Security components		Protected	Extra
		Lambda Layer (ms)	U-Net (ms)	**Total** (ms)	**Overhead**
HybridSN	15.2	+1.2	+3.5	19.9	**+4.7 ms**
3D-CNN	10.5	+0.9	+3.2	14.6	**+4.1 ms**
3D-DL	18.3	+1.2	+3.6	23.1	**+4.8 ms**

Overall, the total extra computational overhead for a fully protected inference request ranges from 4.1 ms (3D-CNN) to 4.8 ms (3D-DL), i.e., merely 4–5 ms across all models. Relative to the inherent network transmission delays in cloud APIs and the heavy spatial-spectral feature extraction of the baseline models, this millisecond-level latency is virtually imperceptible to authorized users. These results demonstrate that our multi-layer defense framework achieves robust, active IP protection without compromising the operational efficiency of the MLaaS platform.

## Discussion

We present a multi-layer active IP protection framework tailored for HSI classification models deployed in cloud MLaaS environments. The framework combines band-aware LSB steganography, class-hiding backdoor watermarking, Lambda-layer authorization, and probability-map ownership encoding. This design addresses three HSI-specific deployment issues: spectral preprocessing may remove ordinary watermarks, visible label inconsistency can expose backdoor triggers in classification maps, and passive ownership verification cannot prevent unauthorized API use before misuse occurs.

Experiments across three benchmark datasets (Indian Pines, PU, and SA) and three spectral-spatial CNN architectures (HybridSN, 3D-CNN, and 3D-DL) show that the protected models retain primary classification fidelity, with no statistically significant OA degradation in repeated trials. The framework achieves authorized user traceability exceeding 99.7% *D*_*uf*_ across all tested models and WM above 97.8%. Robustness tests further show that ownership evidence remains detectable after pruning, distillation, and fine-tuning, but aggressive pruning substantially weakens authorization-related signals; therefore, the evidence should be interpreted as partially detectable rather than fully preserved under aggressive purification. The current study focuses on representative CNN-based HSI classifiers; extending the same protection pipeline to recurrent, Transformer, and Mamba-based HSI models will further clarify its generality across newer architectures.

### Limitations of the study

This study has several limitations. First, the experiments use random pixel-wise train/validation/test splits rather than spatially disjoint block splits. Because spatial autocorrelation in hyperspectral scenes can make pixel-wise protocols optimistic for absolute classification accuracy, our conclusions should be interpreted as paired comparisons between original and protected models under the same split, not as a claim of state-of-the-art HSI classification accuracy.

Second, the empirical validation focuses on three representative spectral-spatial CNN classifiers: HybridSN, 3D-CNN, and 3D-DL. The framework is interface-compatible with classifiers that accept spectral-spatial patches and return class probabilities, including recurrent, Transformer-based, and Mamba-based models. However, these model families may require retuning of the key-sample ratio, confidence threshold, and encoder-decoder input shape. Their full empirical evaluation remains future work.

Third, the robustness results support partial ownership detectability after model purification rather than full preservation of all authorization metrics. Moderate pruning, distillation, and fine-tuning retain detectable watermark evidence, but aggressive 70% pruning substantially weakens ASR, *D*_*u*_, and WM Acc. In addition, LSB-based fingerprint embedding relies on the secrecy of the selected key bands and is less suitable for a known-band, known-algorithm attacker unless replaced by stronger adaptive steganography.

## Resource availability

### Lead contact

Further information and requests for resources should be directed to and will be fulfilled by the lead contact, Hengbo Chen (chenhengbo615@163.com).

### Materials availability

This study did not generate new unique physical materials.

### Data and code availability

• This study used three third-party public hyperspectral benchmark datasets: Indian Pines, PU, and SA. No new primary HSI data were generated. The exact benchmark files analyzed were the corrected image cubes and ground-truth maps customarily distributed as Indian_pines_corrected.mat/Indian_pines_gt.mat, PaviaU.mat/PaviaU_gt.mat, and Salinas_corrected.mat/Salinas_gt.mat. These data resources are listed in the key resources table.
• Source code for key-sample generation, watermark training, authorization evaluation, robustness testing, and trained model configuration files is available from the lead contact upon reasonable request. Public repository accession information will be supplied to the journal before publication if a public code archive is required for production.
• Source data supporting the quantitative results are included in the tables and figures of this paper. Access instructions for the exact third-party benchmark files and any additional information required to reanalyze the data reported in this paper are available from the lead contact upon reasonable request.

## Acknowledgments

This research was supported by the National Natural Science Foundation of China (grant no. 62476013), the Beijing Electronic Science and Technology Institute first-class discipline construction project (grant no. 20260002Z0411), and the Fundamental Research Funds for the Central Universities (grant no. 3282026040).

## Author contributions

H.C. and S.X. conceived the study; H.C. designed the framework and performed the experiments; H.C. and J.Z. implemented the model protection and evaluation pipeline; Y.L. and C.G. contributed to experimental validation and result analysis; S.D. contributed to data organization and manuscript preparation; H.C. wrote the original draft; S.X., J.Z., Y.L., C.G., and S.D. reviewed and edited the manuscript; S.X. and H.C. supervised the project and acquired funding.

## Declaration of interests

The authors declare no competing interests.

## STAR★Methods

### Key resources table

**Table:** 

REAGENT or RESOURCE	SOURCE	IDENTIFIER
Indian Pines hyperspectral dataset	EHU/GIC public HSI benchmark collection	Corrected cube and ground-truth map
Pavia University hyperspectral dataset	EHU/GIC public HSI benchmark collection	Image cube and ground-truth map
Salinas Scene hyperspectral dataset	EHU/GIC public HSI benchmark collection	Corrected cube and ground-truth map
Implementation code and model configuration files	This paper; lead contact	Available upon request; public accession pending
HybridSN classifier architecture	Roy et al.^18^	N/A
3D-CNN classifier architecture	Li et al.^16^	N/A
3D-DL classifier architecture	Hamida et al.^17^	N/A
BS-Nets band-selection method	Cai et al.^9^	N/A
U-Net encoder-decoder architecture	Ronneberger et al.^36^	N/A

### Method details

#### Threat model and security assumptions

We consider an HSI classification model deployed on a cloud-based MLaaS platform, where users interact via black-box APIs. The adversary has two primary goals: (1) *Service Abuse*, attempting to bypass API authorization for illegal use; and (2) *Model Piracy*, attempting to steal and modify the model to obliterate embedded watermarks. Accordingly, we evaluate our defense against two attacker capabilities: **Black-box attackers**, who can only query the API and attempt to forge key samples, and **White-box attackers**, who have illicitly obtained the model parameters (*M*_*em*_) and perform purification attacks (e.g., pruning, distillation, fine-tuning). Crucially, to establish a realistic defense boundary, we assume the cryptographic components (e.g., the user fingerprint database and U-Net decoder weights) and the server-side execution of the Lambda layer remain strictly confidential and secure from the attacker.

#### Framework overview

Our method consists of four phases: key sample generation, model training and deployment, key access control, and user tracking and copyright certification, as illustrated in Figure 1. First, we select base samples from the HSI training set and use steganography to generate key samples with user identity information. Band selection is used to embed information only in key bands, preventing degradation from dimensionality reduction. Second, we assign additional target labels to key samples and inject them into the training set for backdoor watermark embedding. The model owner assigns unique key samples to each authorized user and adds control layers. Class-hiding ensures the target category matches the base sample category, preventing backdoor information leakage. Finally, an encoding network embeds owner identity into classification outputs, enabling remote ownership verification.

The proposed framework is model-agnostic at the interface level: it requires an HSI classifier that accepts spectral-spatial patches and returns class probabilities, but it does not require a particular internal convolutional architecture. We use HybridSN, 3D-CNN, and 3D-DL because they are standard spectral-spatial baselines with different 3D convolutional designs. The same protection pipeline can in principle be attached to recurrent, Transformer, or Mamba-based HSI classifiers,^19^^,^^20^^,^^21^ provided that the deployed model exposes softmax outputs for authorization and ownership encoding. The main model-specific hyperparameters are the key-sample proportion, the confidence threshold in the Lambda layer, and the output shape consumed by the encoder-decoder module.

#### Spectral-domain LSB steganography

We use LSB replacement to embed user fingerprints in hyperspectral images by replacing the least significant bits with message bits. Each base sample contains a unique user fingerprint (e.g., “users Bob”) to identify authorized users.

##### Band selection strategy

Hyperspectral images exhibit issues such as spectral redundancy and irregular data distribution. Consequently, general hyperspectral image classification methods perform dimensionality reduction operations during the preprocessing stage to alleviate the computational and storage burden on training classifiers. However, these operations may compromise embedded identity information, resulting in the failure of user identity management. To ensure effective authorization control and identity management, band selection technology is utilized to embed user identity information exclusively in key bands.

For base sample images of size *S* × *S* × *C*, containing *S* × *S* pixels, where *S* represents the width and height of the image, and *C* represents the number of bands. Base samples are input into the end-to-end BS-Nets^9^ network, which consists of two core modules: (1) a **Band Attention Module (BAM)** that explicitly models nonlinear inter-dependencies between spectral bands via attention mechanisms, addressing the limitation of traditional methods that evaluate bands independently; and (2) a **Reconstruction Network (RecNet)** that restores the original HSI from the selected informative bands, enabling end-to-end training. Treating band selection as a sparse spectral reconstruction task allows the model to learn band weights from full spectral bands to filter and obtain *B* key bands. Band selection is an offline, one-time process conducted by the model owner prior to model training. BS-Nets is trained separately on each dataset (IPINP PU, SA) to accommodate different band counts (*C* = 200, 103, 204, respectively); each dataset uses its own trained BS-Nets to produce dataset-specific *B*_*key*_, ensuring optimal band selection for the spectral characteristics of that scene.

**Key Band Selection Parameter** ***B*:** The number of key bands B is a critical parameter that balances between fingerprint capacity and concealment. Through empirical analysis across three datasets (INP: 200 bands, PU: 103 bands, SA: 204 bands), we determine B as approximately 15%–20% of the total bands C. ands *C*. This selection is based on: (1) *Capacity requirement*: Each fingerprint string (e.g., ”users Bob”) requires approximately 64–128 bits. With *S* × *S* pixels per band and LSB embedding (1 bit per pixel), *B* bands provide *B* × *S* × *S* bits, ensuring sufficient capacity. For typical patch sizes (*S* = 25), *B* = 20 bands provide 12,500 bits, far exceeding requirements. (2) *Concealment requirement*: Selecting too many bands increases the probability of detection. Our experiments show that *B*/*C* < 0.2 maintains spectral angle *θ* < 0.3 radians, ensuring visual imperceptibility. (3) *Security requirement*: The combination space CB should be sufficiently large to resist brute-force attacks. For *C* = 200 and *B* = 30, 20030≈1035, providing strong security guarantees.

##### Multi-band LSB steganographic embedding

The multi-band LSB replacement steganographic algorithm embeds user fingerprints by replacing the least significant bits of key band component values at each pixel. Unlike RGB images, hyperspectral images contain more bands, with each pixel value composed of 16 bits (ranging from 0 to 65535). The diverse bands ensure sufficient pixels for embedding user identity information even after band selection, as shown in Figure 2.

Let $D_{\mathrm{train}}={\left\{\left(x_{i},y_{i}\right)\right\}}_{i=1}^{n}$ denote a benign training set containing *n* samples, where *x*_*i*_ ∈ *X* = {0, …,65535}^*C*×*S*×*S*^ (channel-first format: *C* bands ×*S* height ×*S* width). Base samples $W_{b}={\left\{x_{i}\right\}}_{i=1}^{K}$ constitute a subset of the original training set, where *K* denotes the number of base samples in the subset. The user fingerprint set $FP={\left\{f_{i}\right\}}_{i=1}^{K}$ comprises fingerprints from *K* users, where *f*_*i*_ denotes the fingerprint of the *i*-th user. Key bands containing user identity information replace their corresponding bands in the original base samples to generate hyperspectral key samples $W_{m}={\left\{x_{i}^{\prime }\right\}}_{i=1}^{K}$, where $x_{i}^{\prime }$ denotes the *i*-th key sample. Algorithm 1 provides the pseudocode for this procedure. A schematic diagram illustrating the relationship between base samples and key samples is shown in Figure 3.

Algorithm 1 **Key Sample Generation via Band-Aware LSB Steganography**

**Input:** Base samples $W_{b}={\left\{x_{i}\right\}}_{i=1}^{K}$; fingerprint set $FP={\left\{f_{i}\right\}}_{i=1}^{K}$; BS-Nets for band selection

**Output:** Key samples $W_{m}={\left\{x_{i}^{\prime }\right\}}_{i=1}^{K}$

1: **begin**

2: *B*_*key*_ ←BS-Nets(*W*_*b*_)         ⊳ Select *B* key bands

3: *W*_*m*_ ←∅

4: **for** *i* ∈ {1, …, *K*} **do**

5: $x_{i}^{\prime }\leftarrow \text{LSB\_Embed}\left(x_{i},f_{i},B_{\mathrm{key}}\right)$     ⊳ Embed *f*_*i*_in key bands of *x*_*i*_

6: $W_{m}\leftarrow W_{m}\cup \left\{x_{i}^{\prime }\right\}$

7: **end** **for**

8: **return** *W*_*m*_

9: **end**

##### Security analysis

Steganographic algorithms, when combined with band selection, can control spectral differences between key samples and base samples while also increasing the difficulty of retrieving information locations, thereby enhancing fingerprint concealment. Due to the diversity of hyperspectral image bands, fingerprint-containing bands represent a relatively low proportion of the overall bands, with only model owners aware of the specific band locations, which enhances fingerprint concealment. Attackers selecting *B* key bands from *C* bands in hyperspectral images face a maximum combination number of CB, making brute-force attacks on key samples quite challenging. Furthermore, the values of *C* and *B* are determined by model owners, making it difficult for attackers unfamiliar with the method to forge key samples. Additionally, due to the inherent intra-class spectral variability and inter-class spectral similarity in hyperspectral images and variations in embedded fingerprints among different base samples, collaboration among multiple users becomes challenging, making it difficult for dishonest authorized users to extract regular fingerprint information from key samples.

LSB Steganography Security Discussion: LSB replacement is known to be vulnerable to statistical steganalysis (e.g., RS analysis, *χ*^2^ test) when applied to natural RGB images, as it introduces detectable asymmetry in the histogram of pixel values. In our HSI setting, several factors mitigate this risk: (1) *Band sparsity*: Fingerprints are embedded in only *B* ≪ *C* bands selected by BS-Nets; an attacker without knowledge of *B*_*key*_ must analyze all CB band combinations, making targeted steganalysis computationally infeasible. (2) *High bit depth*: HSI pixels typically use 16-bit values (0–65535); LSB modification alters at most 1/65536 of the dynamic range, yielding negligible statistical deviation compared to 8-bit RGB. (3) *Spectral redundancy*: Natural spectral correlations across bands further obscure LSB-induced artifacts. (4) *Limitation*: Under a known-band, known-algorithm threat model, LSB remains detectable. We therefore recommend combining LSB with band selection secrecy and access control; for higher security requirements, adaptive steganography (e.g., syndrome-trellis codes) could replace plain LSB at the cost of embedding capacity.

#### Backdoor watermark

After generating key samples, we embed an additional class in the DNN as a watermark. Backdoor watermarks in natural images face label consistency problems, which are worse in HSI classification where outputs are classified maps with visible color transformations. We propose a class-hiding method to address this, as shown in Figure 4.

##### Backdoor training set construction

First, backdoor sample training sets are constructed using key samples generated from base samples. Base samples $W_{b}={\left\{x_{i}\right\}}_{i=1}^{K}$ and their corresponding labels *y*_*b*_ form the base sample dataset $D_{b}={\left\{\left(x_{i},y_{b}\right)\right\}}_{i=1}^{K}$. Additional target labels *y*_*t*_(*y*_*t*_∉*Y*) are assigned to generated keys, constituting key sample set $D_{m}={\left\{\left(x_{i}^{\prime },y_{t}\right)|x_{i}^{\prime }=G\left(x_{i}\right),\left(x_{i},y_{b}\right)\in D_{b}\right\}}_{i=1}^{K}$, where *G*: *X* → *X*′ represents the key sample generation method specified by the model owner.

The benign training set *D*_*train*_ and key sample set *D*_*m*_ are combined to form the backdoor training set *D*_*p*_, defined as:

(Equation 2) $$D_{p}=D_{m}\cup D_{\mathrm{train}}$$

where $\gamma =\frac{\left|D_{m}\right|}{\left|D_{\mathrm{train}}\right|}$ represents the key sample proportion ratio. The smaller the value of *γ*, the more covert the backdoor becomes. It is important to note that the added class is supplementary and not one of the classes in the model’s primary classification task. Furthermore, the target label is a representative string that model owners can design flexibly, such as the owner’s name, organization, etc.

**Key Sample Proportion Ratio** ***γ*:** The parameter *γ* critically affects both backdoor effectiveness and model fidelity. Through systematic experiments, we determine the optimal *γ* range as 0.05–0.15 (5%–15% of training samples). This selection is justified by: (1) *Backdoor effectiveness*: When *γ* < 0.05, the backdoor trigger becomes too weak, resulting in ASR <85%. When *γ* > 0.15, the backdoor becomes too prominent, potentially causing detectable artifacts. Our experiments show that *γ* = 0.10 achieves ASR >94% while maintaining classification accuracy within 0.5% of the baseline. (2) *Concealment*: Lower *γ* values enhance backdoor concealment but may compromise trigger reliability. The range 0.05–0.15 provides an optimal trade-off. (3) *Training stability*: Higher *γ* values can cause training instability due to class imbalance. Our selected range ensures stable convergence across all three model architectures (HybridSN, 3D-CNN, 3D-DL).

##### Backdoor model training

Second, model owners perform standard training on deep neural networks (DNNs) using the backdoor training set *D*_*p*_ expressed as follows:

(Equation 3) $$\min_{w}\frac{1}{N+K}\left[\overset{N}{\underset{i=1}{\sum }}L\left(x_{i},y_{i},w\right)+\overset{K}{\underset{j=1}{\sum }}L\left(x_{j}^{\prime },y_{t},w\right)\right]$$

where *w* denotes model parameters and *L* signifies the loss function, such as cross-entropy. Optimization can be solved through backpropagation using stochastic gradient descent. Upon completion of training, backdoor watermarks will be embedded into the classification model, resulting in a backdoor watermarked model *M*_*wm*_. Algorithm 2 provides the pseudocode for the backdoor embedding and class-hiding procedure. During training, DNNs learn mappings from image features to target labels. When making inferences on specific inputs, backdoor DNN *M*_*wm*_ can output specific target classes. It is important to note that embedding backdoors does not introduce new functionality to the model; rather, it adds target categories based on the original classification capabilities.

Algorithm 2 **Backdoor Watermark Embedding with Class-Hiding**

Input: Benign training set *D*_*train*_; key samples *W*_*m*_; base dataset *D*_*b*_ = {(*x*_*i*_, *y*_*b*_)}; target label *y*_*t*_∉*Y*

**Output:** Backdoor model *M*_*wm*_; encrypted model *M*_*em*_ with class-hiding layer

1: **begin**

2: $D_{m}\leftarrow \left\{\left(x_{i}^{\prime },y_{t}\right)|x_{i}^{\prime }\in W_{m},\left(x_{i},y_{b}\right)\in D_{b}\right\}$

3: *D*_*p*_ ← *D*_*train*_ ∪ *D*_*m*_

4: Train DNN on *D*_*p*_ via Eq. (3) to obtain *M*_*wm*_

5: Integrate class-hiding layer: *M*_*em*_(*x*) = *y*_*b*_ if *M*_*wm*_(*x*) = *y*_*t*_, else *M*_*wm*_(*x*)

6: **return** *M*_*em*_

7: **end**

##### Class-hiding mechanism

Finally, a dynamic class-hiding layer is integrated into the deployed model to create the encrypted model *M*_*em*_. Since the backdoor model *M*_*wm*_ natively predicts the explicit target label *y*_*t*_ for key samples, directly outputting *y*_*t*_ would expose the existence of the backdoor. To address this, the class-hiding mechanism operates as a deterministic post-processing mapping layer: when *M*_*wm*_ predicts *y*_*t*_ with high confidence, *M*_*em*_ automatically remaps the output to the ground-truth semantic label *y*_*b*_ of the base sample. This process ensures that authorized users do not obtain any information about the backdoor target category during normal usage, preserving visual label consistency in the HSI classification maps and effectively concealing the backdoor from adversarial observation.

#### Lambda authorization layer

To ensure that models grant access permissions solely to authorized users in large-scale user distribution systems while restricting unauthorized access and illegal usage, this paper employs a user identity authentication scheme that achieves fine-grained authorization control by assigning specific key samples containing user identity information to authorized users. It is important to note that this paper assumes that keys are transmitted through secure and confidential communication.

##### Lambda layer-based access control

To implement authorization control, this paper constructs a Lambda layer-based access control mechanism based on specific mapping relationships between key samples and target labels, incorporating it at the end of encrypted models. Authorized users are required to submit key samples to MLaaS for identity verification before gaining effective inference capabilities from DNN models.

The Lambda layer is implemented as a custom layer that intercepts model predictions and performs real-time authorization checks. The control layer involves several conditions and tensor operations, implemented specifically as follows:

**Confidence Verification:** For an input key sample $x_{i}^{\prime }$, the Lambda layer first computes the prediction confidence $P_{\mathrm{conf}}\left(x_{i}^{\prime }\right)=\max\left(\text{softmax}\left(M_{em}\left(x_{i}^{\prime }\right)\right)\right)$ where *M*_*em*_ denotes the encrypted model. The error *E*_*c*_ between this confidence and a predefined threshold *τ*_*conf*_ (typically set to 0.95) is calculated as:

(Equation 4) $$E_{c}=|P_{\mathrm{conf}}\left(x_{i}^{\prime }\right)-\tau_{\mathrm{conf}}|$$

This threshold *τ*_*conf*_ is empirically determined based on the model’s typical confidence distribution for legitimate key samples, ensuring that authorized users’ key samples produce high-confidence predictions while unauthorized inputs fail this check.

**User Identity Extraction:** The Lambda layer extracts user identity information from key samples using the LSB extraction algorithm. For each key sample $x_{i}^{\prime }$, the layer extracts the embedded fingerprint $f_{\mathrm{extracted}}=\text{LSB\_Extraction}\left(x_{i}^{\prime },B_{\mathrm{key}}\right)$ where *B*_*key*_ represents the set of key bands selected during the band selection process. The extraction process involves: (1) identifying key bands based on the band selection mask; (2) reading the least significant bits from pixels in these bands; (3) reconstructing the binary fingerprint string through concatenation.

**Authorization Decision:** A user is identified as authorized only if both conditions are satisfied: (1) *E*_*c*_ < *ϵ*_*tol*_ where *ϵ*_*tol*_ is a tolerance threshold (typically 0.05), and (2) *f*_*extracted*_ ∈ *FP* where *FP* is the authorized fingerprint database maintained by the model owner. If either condition fails, the user is classified as unauthorized.

**Output Control:** For authorized users, the Lambda layer passes through the original model predictions $M_{em}\left(x_{i}^{\prime }\right)$ to the encoder–decoder network for watermark embedding. For unauthorized users, the layer outputs random prediction results ${\tilde{y}}_{\mathrm{random}}$ sampled from a uniform distribution over the label space, effectively preventing unauthorized access while maintaining API response consistency to avoid revealing the protection mechanism.

##### Identity verification process

The entire authorization and output control process orchestrated by the Lambda layer can be formalized as a conditional function

(Equation 5) $$A\left(x_{i}^{\prime }\right)=\left\{\begin{array}{cc} E\left(M_{em}\left(x_{i}^{\prime }\right),f_{\mathrm{owner}}\right), & \text{if}\;E_{c}<\epsilon_{\mathrm{tol}}\wedge f_{\mathrm{extracted}}\in FP\\ {\tilde{y}}_{\mathrm{random}}, & \text{otherwise} \end{array}\right)$$

where E represents the subsequent encoder-decoder network used to imprint the owner’s fingerprint *f*_*owner*_ into the classification output, and ${\tilde{y}}_{\mathrm{random}}$ denotes the random prediction (e.g., uniformly sampled label or probability distribution over the label space) returned to unauthorized users. This mathematical formalization guarantees that the decrypted predictions are strictly accessible only when both the confidence criteria and user identity authentication are simultaneously satisfied. Algorithm 3 provides the pseudocode for the complete authorization flow.

Algorithm 3 **Lambda layer authorization control**

**Input:** Input sample $x_{i}^{\prime }$; encrypted model *M*_*em*_; fingerprint database *FP*; key bands *B*_*key*_

**Output:** Authorized output $E\left(M_{em}\left(x_{i}^{\prime }\right),f_{\mathrm{owner}}\right)$ or random output ${\tilde{y}}_{\mathrm{random}}$

1: **begin**

2: $P_{\mathrm{conf}}\leftarrow \max\left(\text{softmax}\left(M_{em}\left(x_{i}^{\prime }\right)\right)\right)$

3: *E*_*c*_ ←|*P*_*conf*_ − *τ*_*conf*_|

4: $f_{\mathrm{extracted}}\leftarrow \text{LSB\_Extraction}\left(x_{i}^{\prime },B_{\mathrm{key}}\right)$

5: **if** *E*_*c*_ < *ϵ*_*tol*_ **and** *f*_*extracted*_ ∈ *FP* **then**

6: **return** $E\left(M_{em}\left(x_{i}^{\prime }\right),f_{\mathrm{owner}}\right)$

7: **else**

8: **return** ${\tilde{y}}_{\mathrm{random}}$     ⊳ Unauthorized: output random prediction

9: **end** **if**

10: **end**

##### User traceability tracking

Furthermore, this method enables model owners to trace dishonest authorized users by retrieving authorized user information from key samples to uphold ownership rights. Owners can extract key bands from illegally distributed or copied key samples to facilitate steganographic detection. If authorized user identity information is successfully detected from key samples, model owners can promptly implement accountability measures against the owners of the key samples to prevent unnecessary consequences.

#### Model IP authentication

Most existing deep models are deployed in the cloud or offered as API services. In these scenarios, although attackers cannot employ traditional model attacks, the risk of direct model theft by internal personnel persists. Current active IP protection methods excessively depend on key sample design, making it challenging to differentiate between model owners and authorized users. This situation leads to issues such as collusion attacks and excessively high security requirements for key samples.

##### Encoder–decoder network design

We embed ownership fingerprints seamlessly into the spatial probability maps generated by the classification model, rather than the discrete one-hot labels. HSI classification models typically operate in a patch-based manner: each input patch of size *S* × *S* × *C* (e.g., 25 × 25 × 103 for PU) yields a per-patch class probability vector of dimension |*Y*| (number of classes). For ownership verification, we collect the softmax outputs from multiple key samples; the U-Net encoder takes these probability vectors as input. The input tensor has shape *N* ×|*Y*|× *H* × *W*, where *N* is the batch size and *H* × *W* is the spatial resolution of the classification output. For patch-wise classification (as in HybridSN, 3D-CNN, 3D-DL), each patch produces one prediction, so *H* = *W* = 1 and the effective input is *N* ×|*Y*|× 1 × 1; the U-Net operates on this representation to embed the owner fingerprint while preserving the probability distribution. Inspired by deep invisible watermarking,^37^ we adapt the U-Net^36^ architecture—originally designed for spatial pixel manipulation in StegaStamp^38^—to function as our probability map encoder. The U-Net encoder-decoder design comprises: (1) **Encoder path**: a contracting path with repeated 3 × 3 convolutions, ReLU activations, and 2 × 2 max-pooling that progressively downsamples the input and doubles the number of feature channels at each stage; (2) **Bottleneck**: the lowest-resolution representation; (3) **Decoder path**: a symmetric expanding path with upsampling and skip connections to restore the original dimensions; and (4) **Output layer**: a 1 × 1 convolution that produces the watermarked probability map with the same dimensions as the input. This design ensures that watermark embedding introduces minimal distortion while enabling reliable extraction.

##### Watermark embedding process

Model owners incorporate encoding networks as post-processing modules into encrypted models while retaining decoder networks for subsequent IP authentication tasks. Classification outputs from encrypted models are fed into encoding networks, which embed pre-set owner fingerprints into the original classification outputs. This process generates specific classification outputs visually consistent with the original results, returning them as final results to authorized users through APIs.

##### Ownership verification protocol

Owners submit key samples to suspicious models *M*_*s*_ through APIs to obtain remote usage rights and use retained decoder networks to decode the classification outputs from these potentially compromised models. If the extracted information matches the model owner fingerprints, model *M*_*s*_ can be identified as a pirated model, triggering subsequent accountability and infringement processing measures. Algorithm 4 provides the pseudocode for the ownership verification protocol. This method addresses collusion attacks and improves authentication reliability.

Algorithm 4 **Remote ownership verification protocol**

**Input:** Suspicious model *M*_*s*_ (API access); key samples *W*_*m*_; decoder D; owner fingerprint *f*_*owner*_

**Output:** Verification result: pirated or benign

1: **begin**

2: Submit key samples *W*_*m*_ to *M*_*s*_ via API to obtain outputs {*y*_*i*_}

3: $f_{\mathrm{extracted}}\leftarrow D\left(\left\{y_{i}\right\}\right)$      ⊳ Decode fingerprint from outputs

4: **if** *f*_*extracted*_ = *f*_*owner*_ **then**

5: **return** “Pirated”        ⊳ Ownership confirmed

6: **else**

7: **return** “Benign”

8: **end** **if**

9: **end**

#### Experimental datasets

We use three hyperspectral datasets: Indian Pines (INP), Pavia University (PU), and Salinas Scene (SA), covering rural, urban, and agricultural scenarios. Table 12 summarizes the datasets.

1) Indian Pines: This image was captured by the Airborne Visible Infrared Imaging Spectrometer (AVIRIS) sensor from NASA’s Jet Propulsion Laboratory in the pine forest region of northwestern Indiana, USA. The image size is 145 × 145 pixels, containing 200 spectral bands in the wavelength range of 0.4–2.5 *μ*m. The image contains 10,249 labeled pixels, which belong to 16 forest and cropland cover categories.
2) Pavia University: This image was captured by the Reflective Optics Spectrographic Imaging System (ROSIS) from the German Aerospace Center over the University of Pavia, Italy. The pixel size is 610 × 340 pixels, with 103 spectral bands in the wavelength range of 0.4–0.9 *μ*m. A total of 42,776 pixels are labeled in the image, which belong to 9 urban land cover categories.
3) Salinas Scene: This image was captured by AVIRIS over the Salinas Valley in California, USA. The pixel size is 512 × 217 pixels, containing 204 bands in the wavelength range of 0.4–2.5 *μ*m. The image contains 54,129 labeled pixels across 16 categories, most of which belong to crops.

**Table 12:** Basic information on the three hyperspectral datasets

Dataset	Classes	Image size	Bands	Range (nm)	Resolution (m)
Pavia University	9	610 × 340	103	430–860	1.3
Indian Pines	16	145 × 145	200	400–2,500	20
Salinas	16	512 × 217	204	400–2,500	3.7

#### Experimental settings

1) Experimental Environment: All experiments were conducted on a computer equipped with an AMD R7-5800H processor, 16 GB of RAM, and an NVIDIA GeForce RTX 4090 graphics card.
2) Deep Learning Models: This paper evaluates the effectiveness of the multi-layer active intellectual property protection framework using three HSI classification methods based on DL:
  HybridSN^18^: Utilizes a 3D-CNN to represent the joint spatial-spectral features of spectral bands and further learns abstract spatial features through a 2D-CNN. The original model employs the Adam optimizer with a learning rate of 1e−3 for 100 epochs. The backdoor model is trained for 150 epochs with an Adam learning rate of 5e−4 and a weight decay of 1e−6.
  3D-CNN^16^: Takes hyperspectral cube data blocks as input and utilizes 3D convolutional neural networks for spatial-spectral fusion classification. The original model employs the SGD optimizer for 100 epochs with a learning rate of 1e−2 and a weight decay of 5e−4. Fine-tuning is performed on the backdoor training set for 50 epochs.
  3D-DL^17^: Utilizes a 3D deep learning framework for the joint processing of spectral and spatial information in hyperspectral remote sensing images. It employs the SGD optimizer for 200 epochs with a learning rate of 1e−2, a weight decay of 5e−4, and a momentum parameter of 0.9.
3) Band Selection and BS-Nets: BS-Nets is trained separately on each dataset (INP, PU, SA) using the spectral reconstruction objective (reconstructing the full HSI from selected bands). Training is performed on the same training split used for the classification model. Each dataset’s BS-Nets is trained for 50 epochs with the Adam optimizer (lr = 1e−3). The number of key bands *B* is set to 15–20% of *C* per dataset.
4) Data Splitting, Key Sample Selection, and Reproducibility: For each dataset, labeled pixels are first split into training, validation, and test partitions without overlap. Unless otherwise specified, 20% of labeled pixels are used for training, 10% of the training partition is reserved for validation and hyperparameter selection, and the remaining labeled pixels are used for testing. The split is a random pixel-wise split rather than a spatially disjoint block split. We explicitly acknowledge that spatial autocorrelation in HSI scenes can make random pixel-wise protocols optimistic for absolute classification accuracy; therefore, our conclusions focus on paired relative comparisons between original and protected models under the same split, rather than on claiming state-of-the-art HSI classification accuracy. The same split is used for the original and protected versions of each classifier to ensure paired comparison. Key base samples are selected only from the training partition and are never drawn from validation or test pixels. For each authorized user, one or more base samples are converted into key samples by embedding the user fingerprint in the selected key bands. Experiments are repeated 10 times with different random seeds controlling data partitioning, weight initialization, and key-sample selection; all reported values are mean ± standard deviation. We do not mix training and test pixels during key generation, watermark training, or ownership verification.

### Quantification and statistical analysis

To comprehensively assess both classification utility and IP protection efficacy, we employ multiple evaluation metrics. For the primary classification task, we utilize Overall Accuracy (OA), Average Accuracy (AA), and the Kappa coefficient (*κ*). To evaluate the backdoor watermark, we measure the Attack Success Rate (ASR) on backdoor-triggered samples, where an ASR > 90% typically indicates an effective backdoor. For steganography invisibility, we use Peak Signal-to-Noise Ratio (PSNR) and Structural Similarity Index (SSIM). For authorization and traceability, we report the Watermark Detection Accuracy (WM Acc), User Detection Rate (Du Rate), False Accept Rate (FAR), and False Reject Rate (FRR). Because the observed utility differences between original and protected models are small, we additionally perform two-sided Welch’s *t*-tests on OA values from the 10 repeated runs for each dataset-model pair. At the 0.05 significance level, none of the original-versus-protected OA differences are statistically significant (all *p* > 0.20; the smallest *p* is 0.209 for HybridSN on Indian Pines). This supports the claim that the proposed protection mechanism does not introduce a statistically detectable loss in primary classification accuracy under the tested settings.
